# Per- and polyfluoroalkyl substances (PFASs) in contaminated coastal marine waters of the Saudi Arabian Red Sea: a baseline study

**DOI:** 10.1007/s11356-020-09897-5

**Published:** 2020-09-07

**Authors:** Aasim M. Ali, Christopher P. Higgins, Walied M. Alarif, Sultan S. Al-Lihaibi, Mohammed Ghandourah, Roland Kallenborn

**Affiliations:** 1 Section of Contaminants and Biohazards, Institute of Marine Research (IMR), P.O 1870 Nordnes, NO-5817 Bergen, Norway; 2 Department of Civil & Environmental Engineering, Colorado School of Mines, 1500 Illinois Street, Golden, CO 80401 USA; 3 Department of Marine Chemistry, Faculty of Marine Sciences, King Abdulaziz University, PO Box 80207, Jeddah, 21589 Saudi Arabia; 4 Arctic Technology Department (AT), University Centre in Svalbard (UNIS), P.O. Box 156, Longyearbyen, Svalbard Norway; 5 Faculty of Chemistry, Biotechnology and Food Science (KBM), Norwegian University of Life Sciences (NMBU), P.O. Box 5003, Christian M. Falsen veg 1, No-1432, Ås, Norway

**Keywords:** Emerging contaminants, LC-MS/MS, SPE, Fluorotelomer sulfonate, Sewage discharge, Fire fighting

## Abstract

Per- and polyfluoroalkyl substances (PFASs) are today considered important constituents of the continuously growing substance group of persistent contaminants of emerging environmental concern (PCEC). Here, we report for the first time the concentrations of 12 relevant PFASs in 28 marine water samples from the Saudi Arabian coastal waters of the Red Sea. The sum levels of 12 PFASs (Σ_12_ PFAS) in surface seawater ranged from <LOQ to 956 ngL^−1^. For the reference background site of this study, Σ_12_ PFAS levels ranged from <LOQ to 10.9 ng/L. The highest PFAS levels have been found in Al-Arbaeen and Al-Shabab, two lagoons continuously receiving treated sewage effluents. PFHxA, PFHxS, and 6:2 FTS were the most prevalent PFASs with relatively high concentrations. Discharge of municipal and industrial wastewaters is considered an important source of PFASs. The pattern of PFASs observed here suggests that the usage of PFAS-containing aqueous film-forming foams (AFFFs) is a potential additional source for these compounds in Al-Arbaeen and Al-Shabab lagoons. However, a systematic elucidation of local PFASs sources is needed. Contamination of the Red Sea waters with PFASs poses a potential imminent risk to the marine environment of the Red Sea and ultimately may even affect the health of human consumers through the consumption of local seafood.

**Electronic supplementary material:**

The online version of this article (10.1007/s11356-020-09897-5) contains supplementary material, which is available to authorized users.

## Introduction

The occurrence of per- and polyfluoroalkyl substances (PFASs, C_n_F_2n + 1_-R) in the aquatic environment is one of the emerging issues in environmental chemistry and risk assessment (Ahrens 2011). It was estimated that > 3000 PFASs are or have been currently on the global market from which 2060 are intentionally manufactured (KemI 2015). Therefore, a wide range of emerging PFASs (e.g., short-chain PFAAs, F-53B, and PAPs) are being detected (Gebbink et al. 2016). PFASs comprise a diverse group of more than 3000 synthetic persistent organic pollutants (Wang et al. 2017). The presence of a perfluoroalkyl moiety (C_n_F_2n + 1_), which contains the extremely strong and stable C-F bond, imparts unique and important properties to PFASs (e.g., thermal stability, higher surface activity at very low concentrations, hydrophobic and lipophobic nature) (Kissa 2001). These unique chemical features lead to many applications of PFASs (e.g., food packaging and aqueous film-forming foams (AFFFs) for fire protection purposes) (Taylor 1999). Numerous additional applications have been described earlier (Posner 2012). Of all PFASs, the perfluoroalkyl sulfonates (PFSAs) and the perfluoroalkyl carboxylates (PFCAs) are the most widely studied (Wang et al. 2017), though there has been an increasing focus on their perfluoroalkyl acid precursors (PFAA-precursors) as well (Dimzon et al. 2017; Li et al. 2018; Makey et al. 2017; Rewerts et al. 2018; Yu et al. 2018).

Compared with their short-chain analogs, long-chain PFSAs (C_n_F_2n + 1_SO_3_H with *n* ≥ 6) and PFCAs (C_n_F_2n + 1_COOH with *n* ≥ 7) have been shown to be more bioaccumulative (Conder et al. 2008; Martin et al. 2003a; Martin et al. 2003b; Olsen et al. 2009). In addition, precursor compounds which can be transformed into PFCAs and PFSAs during environmental processes continue to be produced and released into the environment (Benskin et al. 2012). While the bioaccumulation of some precursors has recently been observed (Asher et al. 2012; Langberg et al. 2019; Reiner et al. 2011), exposure to some precursors has also resulted in metabolic conversion and accumulation of PFCAs and/or PFSAs (D’eon et al. 2011; Fu et al. 2015; McDonough et al. 2020; Rhoads et al. 2008; Xie et al. 2009).

PFASs are continuously released into the environment from various point sources (e.g., sewage treatment plants, application of AFFF in firefighting, industrial installations, shipping, and transportation), and nonpoint sources (e.g., atmospheric deposition, ocean currents) (Ahrens and Bundschuh 2014). As a consequence of this widespread use and application of these substances and their resulting emissions, the environmental presence of a broad range of PFASs is today considered ubiquitous. Several studies report PFASs in drinking waters (Banzhaf et al. 2017; Gebbink et al. 2017; Gellrich et al. 2013; Hu et al. 2016; Kaboré et al. 2018; Lange et al. 2007; Scher et al. 2018; Thomaidi et al. 2020), in seawater (Cai et al. 2012; Cai et al. 2011; Chen et al. 2016; Kallenborn 2004; Lee et al. 2020; Van de Vijver et al. 2007), in sediments (Chen et al. 2016; Munoz et al. 2017a; Munoz et al. 2017b; Pan et al. 2020; Pignotti et al. 2017; White et al. 2015), and in biota (Fair et al. 2019; Suominen et al. 2011; Taylor et al. 2018; Van der Schyff et al. 2020). Furthermore, these substances have been measured in human samples worldwide (Guzmàn et al. 2016; Kannan et al. 2004; Liu et al. 2010; Olsen et al. 2017; Wang et al. 2018). This led to the addition of two PFASs, namely perfluorooctane sulfonate (PFOS) and perfluorooctanoic acid (PFOA) to the Stockholm Convention list of persistent organic pollutants (POPs) in May 2009 and 2019, respectively. However, alternative short-chain products based on per- and poly-fluorinated ethers, perfluorobutane sulfonate (PFBS), and C_6_ fluorotelomer sulfonate (6:2 FTS) raw materials are still produced and applied (Wang et al. 2013). 6:2 FTS derivatives are now being applied in AFFF as substitutes for PFOS and PFOA (Cheremisinoff 2016), though they have also been used historically in some AFFF formulations (Schultz et al. 2004).

Although concentrations of individual PFASs may often be too low to cause hazardous effects, the occurrence of their mixtures in combination with long-term chronic/ sub-chronic exposure can be of significant environmental concern (Ahrens and Bundschuh 2014). Data regarding the ecotoxicological effects of PFASs are, however, still insufficient. A few studies report on the potential adverse effects of PFASs on marine organisms, wildlife, and humans (Alexander and Olsen 2007; Keller et al. 2012; Latała et al. 2009; Lau et al. 2007). At relatively high concentrations, acute toxicity of seven PFASs was reported on two Cladocera (Ding et al. 2012). Zheng et al (2012) observed potential effects on the development of zebrafish embryos under controlled laboratory conditions. Yue Hu et al. (2003) reported that PFOS exposure may even increase the cell membrane fluidity in fish.

The increasing environmental concern regarding the detection, fate, and effects of PFASs has led to the development of a wide spectrum of quantitative analytical methods aiming at determining PFASs at trace levels in different environmental matrices. Solid-phase extraction (SPE) is considered one of the preferred extraction and clean-up methods for PFASs’ quantification in aqueous samples (Van Leeuwen and De Boer 2007). Liquid chromatography coupled to tandem mass spectrometry (LC–QqQ) using selected and multiple reaction monitoring mode (SRM and MRM, respectively) is the current standard quantification technique for PFASs in environmental samples due to its high sensitivity and selectivity (Lacina et al. 2011).

The detected profile of PFASs depends on sources and environmental ambient conditions. Therefore, a comprehensive pattern evaluation may contribute to the complete understanding of local PFAS sources, possible transport pathways, and associated exposure risks for humans and the environment as well. Until today, there are, however, no known scientific reports dealing with the overall presence of PFASs in the marine coastal environment of the Saudi Arabia coast. Thus, the current study reports, for the first time, the levels of selected PFASs in the Eastern coastal waters of the Red Sea and provides conclusions and recommendations for future research and monitoring priorities.

## Material and methods

### Chemicals

Acetonitrile and methanol (MeOH, HPLC grade) were purchased from VWR (West Chester, PA, USA). Reagent-grade ammonium acetate (CH_3_COONH_4_) was purchased from VWR (West Chester, PA, USA); hydrochloric acid (HCl), ascorbic acid, and disodium ethylene diamine tetra acetate (Na_2_EDTA) were purchased from Sigma-Aldrich (Al-Khobar, Saudi Arabia). The water used was ultrapure water produced by a Milli-Q water purification system (Millipore, Bedford, MA, USA).

### Target compounds

The selected 13 PFASs investigated in this study were purchased from Wellington Laboratories (Guelph, ON, Canada) including nine PFCAs (C4–6, 8–13) (three PFSAs (C4, 6, 8), and perfluorooctane sulfonamide (FOSA) and one fluorotelomer sulfonate (6:2 FTS) (please refer to Table S1 in the Supplementary Material (SM) for detailed information on names and properties).

### Sampling area

The surface water of the Red Sea is characterized by relatively high temperatures (up to 34 °C) and high salinity (up to 41 ‰) (Rasul and Stewart 2015). There are two wind-driven water currents in the Red Sea. During the summer, surface current flows toward the south; and during the winter, surface water current flows toward the north (Rasul and Stewart 2015). The Red Sea coastal environments host over 200 coral species. These corals are inhabited by 3000 diverse species of invertebrate, algae, and fish (Goren and Dor 1994; Rasul and Stewart 2015). Moreover, the Red Sea is an economically important region for all surrounding nations due to its industrial resources as well as the advanced tourism and recreational activities in coastal zones (Al-Farawati et al. 2019).

**Fig. 1:**
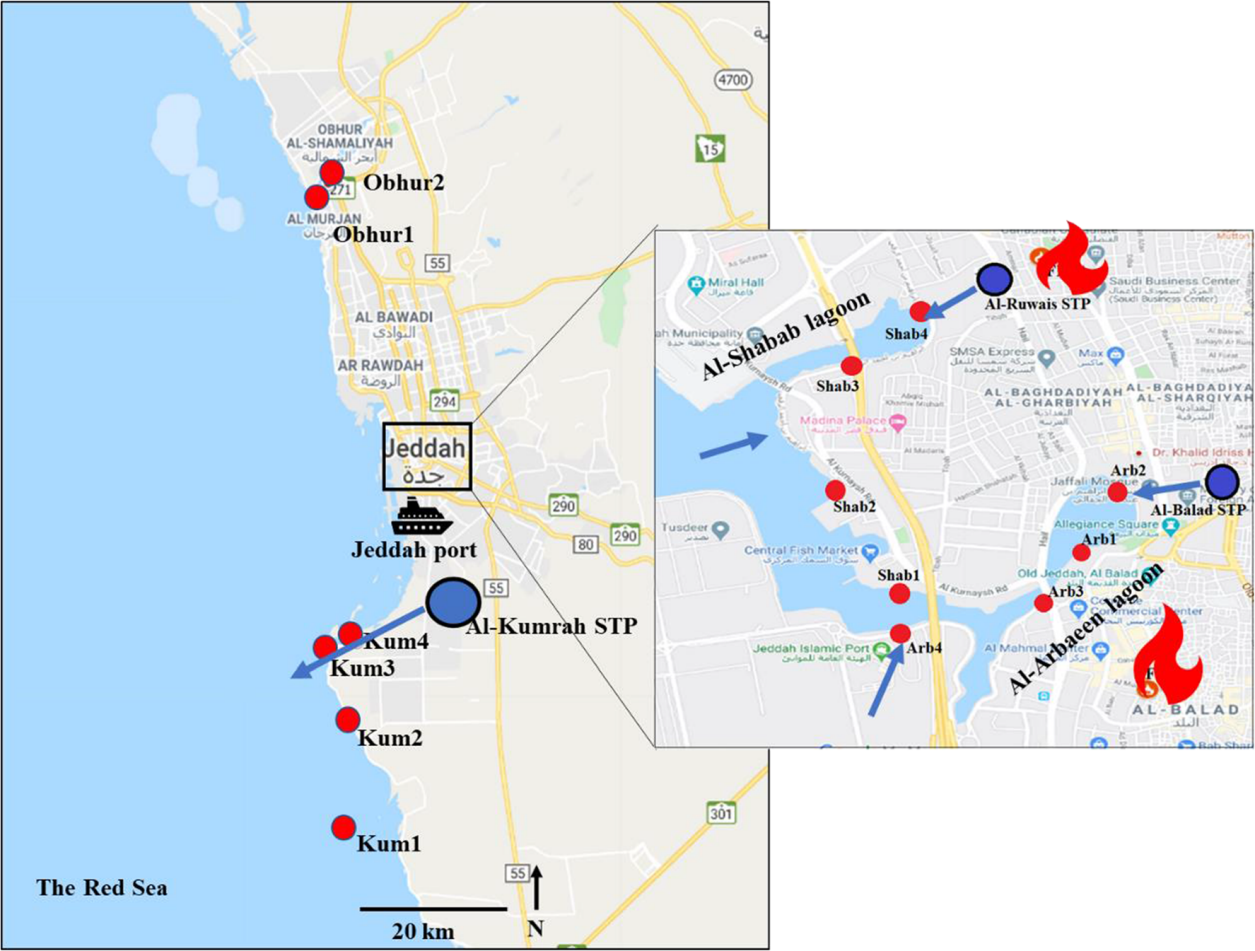
Location map (source google map) showing the sampling sites of the current study. Red solid circles are actually sampling locations. Discharge points of three sewage treatment plants (STP) are depicted by the blue circles: Al-Khumrah, Al-Balad, and Al-Ruwais. The two firefighting stations are shown using fire symbols. Blue arrows indicate positions effluents discharge points

The Red Sea environment, however, is vulnerable to pollution mostly emitted by ship-based transportation. The Red Sea is directly connected to the Indian Ocean. After the installation of the Suez channel in 1869, the regions host one of the most used shipping routes on earth. Furthermore, its coasts are subjected to various commercial activities such as tourist resorts, fishing, and desalination plants (Rasul and Stewart 2015). In the Kingdom of Saudi Arabia (KSA), more than 50% of wastewater is discharged without treatment (Qadir et al. 2010), and the remaining wastewater undergoes secondary treatment (Al-Jassim et al. 2015). Jeddah is a coastal city in the south of KSA of 4 million inhabitants. Jeddah hosts various industrial activities (e.g., oil refineries, food conservation, and canning facilities). Additionally, Jeddah seaport is one of the most important ports along the Red Sea coast. Due to the high degree of industrial and municipal activities in the region, the Red Sea ecosystems around Jeddah has been significantly influenced by the discharge of partially treated municipal and industrial sewage into the coastal marine environments (Ziegler et al. 2016). Al Arbaeen and Al Shabab (Fig. 1) are coastal lagoons located close to Jeddah city center. Al-Shabab lagoon has an elongated shape that favors water exchange with the open sea, while the T-shape of Al-Arbaeen lagoon restricts its water exchange with the open water (El Sayed et al. 2015). For decades, these lagoons received about 100,000 m^3^/day of treated or untreated sewage (El Sayed et al. 2015). In 2002, sewage discharge was officially stopped into the two lagoons with a subsequent implementation of an environmental rehabilitation program. However, field observations and analyses results of hydrochemical parameters and emerging pollutants indicate that the lagoons are still suffering from sewage discharges, particularly the Al-Arbaeen lagoon ecosystem (Al-Lihaibi et al. 2019; Ali et al. 2017; Ali et al. 2018; El Sayed et al. 2015). The effluents discharged into Al-Arbaeen and Al-Shabab lagoons are mainly stemming from the Al-Balad and Al-Ruwais sewage treatment plants (STPs), respectively (Al-Farawati et al. 2019). Moreover, firefighting stations serving the area around Al-Arbaeen and Al-Shabab were identified ca. 400 m from the lagoons as depicted in Fig. 1, representing additional potential sources of PFASs.

In contrast, Al-Khumrah, located in the Southern Corniche of the city, represents the most important STP in Jeddah, delivering around 250,000 m^3^ day^−1^ of tertiary treated effluent to the coast (Al-Farawati et al. 2019). Al-Khumrah STP receives also discharge from industrial installations in the region. Thus, Al-Arbaeen and Al-Shabab receive partially treated domestic sewage in addition to discharges from the firefighting stations, while coastal water around Al-Khumrah STP receives tertiary treated industrial sewage.

Surface seawater was collected from the main effluent dominated sites (Al-Arbaeen, Al-Shabab, and Al-Khumrah) along the Jeddah coast (Fig. 1). Additionally, Sharm Obhur is considered a reference site representing background levels of PFASs in the coastal water off Jeddah. However, there is an activity and beaches around Obhur. The sites shown in Fig. 1 were selected for sampling: four sites at Al-Arbaeen lagoon (Arb 1, Arb 2, Arb 3, and Arb 4) where two samples (about 10 m apart) were collected from each site; four sites at Al-Shabab lagoon (Shab 1, Shab 2, Shab 3, and Shab 4) where two samples (about 10 m apart) were collected from each site (except one sample at Shab 4); four sites at Al-Khumrah (Kum1, Kum2, Kum3, and Kum4) where two samples were collected from each site (except Kum3 where three samples, about 10 m apart, were collected); and two sites at Sharm Obhur (Obhur1 and Obhur2) where two samples (about 10 m apart) were collected from each site, leading to total samples of 28.

### Sample preparation

Surface water samples (about 1 m depth) were collected in Dec. 10–11, 2018, in 1-L gas-tight polyethylene bottles. To remove any chlorine from effluent samples, a total of 4 mL from an aqueous solution containing ascorbic acid (25 mg/L) and Na_2_EDTA (5 g/L) was added to each 1-L water sample (Batt et al. 2008). The pH of each sample was adjusted to 3–4 using 10% HCl solution. The samples were then filtered with GF/C microfiber filters (Whatman Inc., Clifton, NJ). Finally, 20 μL of the 500 ng/mL (each compound) internal standards (ISTDs) solution was added (list of ISTDs can be found in Table S3). As the Red Sea water contains high salt content which causes a signal suppression, samples were extracted and cleaned up by solid-phase extraction (SPE) using Waters Oasis® 500 mg HLB cartridges (Waters, Milford, MA, USA) instead of the common Weak Anion Exchange (WAX) cartridges. The HLB cartridges were placed on a 20-position vacuum Manifold (Supelco, Bellefonte, USA). HLB cartridges were conditioned by 6 mL of methanol followed by 6 mL of water, and then samples were loaded on the cartridges at 1–3 drops/s rate through a polypropylene tubing (o. d. 1/8″) connected to the cartridges by reservoir adapters. The SPE cartridges were washed with 4 mL of 5% MeOH in water. After drying under a vacuum, elution was conducted using 6 mL of methanol in 15-mL polypropylene tubes. The collected eluent was dried under nitrogen gas (6.0 quality, AGA, Porsgrunn, Norway) at 37 °C using a Reacti-Therm III evaporating unit (Thermo Fisher Scientific Inc., Rockford, USA). After the addition of recovery standard [^13^C_8_]-PFOA (500 ng/mL, 40 μL), each sample residue was dissolved in 960 μL of acetonitrile-water (20:80, v/v) with the aid of a vortexing mixer. The samples were filtered by 1.5-mL Costar Spin-x tubes (0.2 μm Nylon, Corning Inc., Corning, NY) and transferred to polypropylene autosampler vials for LC–QqQ analysis.

### Instrumental analysis

Ten microliters of each sample was injected on a Zorbax Eclipse plus C18 RRHD (2.1 × 100 mm, 1.8 μm, Agilent, Palo Alto, USA) combined with a Guard Cartridge (4 μm × 3.0 mm ID) for liquid chromatographic separation at isothermal 30 °C. The mobile phase solvents were water with 5 mM ammonium acetate content (A) and pure MeOH (B) with a flow rate of 0.2 mL/min. The initial mobile phase proportion was 15% (A) and held for 5 min. B was then linearly increased to 99% over 5 min and held for 7 min. B was then linearly changed to 1% at a flow of 1.0 mL/min until the end of the quantitative analysis (total run = 26 min.). Detection and quantification of PFASs were conducted on the Agilent 6460 series triple quadrupole tandem mass spectrometer (QqQ) (Agilent, Santa Clara, USA) equipped with the Jet Stream electrospray ionization (AJS-ESI) source. Selected and multiple reaction monitoring modes (SRM and MRM, respectively) were performed in the negative ion mode. ESI characteristics and MRM transitions are given in Tables S2 and S3-S4, respectively.

### Control of background contamination

A variety of potential PFAS contamination sources are commonly found in commercial LC systems and solvents. Therefore, in this study, a delay column (Agilent Eclipse Plus C18, 4.6 × 50 mm, 3.5 μm) was installed after the mixing valve and before the autosampler to trap PFAS contaminations in the pump system (Powley et al. 2008). Furthermore, field and laboratory blank samples (clean deionized water) were included in the quality control protocols and prepared for quantification in a manner identical to field-collected samples. Methanol was injected after every 10th sample. Furthermore, all direct contact with commercial products containing fluoropolymers, e.g., polytetrafluoroethylene (PTFE), was avoided. Blank samples were below detection limits. Therefore, no blank correction of the results was required.

### Method validation and quality control

To determine the concentration of PFASs in surface water samples collected from the Saudi Red Sea, a multi-component quantification method was optimized and validated. A previously published method by Yamashita et al. (2004) was adopted and modified for the purpose of this study (see “Sampling area” to “Control of background contamination” sections).

Instrument limit of detection (ILOD) and limit of quantification (LOQ) were determined as the amount of the PFAS dissolved in methanol that gave S/*N* = 3 and 10, respectively. For the determination of the method detection limit (MDL), seawater samples spiked near the expected MDL were taken through the entire analytical method. MDLs were determined as the amount of PFAS spiked in seawater that gave S/*N* = 3. Squared coefficient of determination (*R*^2^) for all compound calibration curves were > 0.99, confirming an acceptable linearity range over four orders of magnitude. Furthermore, the linear calibration curves for the internal standards applied for quantification were prepared using four dilution steps (5, 25, 50, 60 ng/mL) and applied [^13^C_8_]-PFOA as a recovery standard. Correlation coefficients for all ISTDs were found > 0.99. The relative method recovery (%) was calculated using seawater sample collected from a depth of 60 m from the Norwegian Oslofjord (south of Drøbak, Norway). Four replicates samples (1 L) were spiked with all native compounds at 50 ng/L and all ISTDs at a concentration of 25 ng/L. All spiked samples were prepared according to the sample preparation and quantification protocol for PFASs developed here for aqueous samples. HPLC-QqQ data were processed with Agilent through the MassHunter Workstation software package (Qualitative and Quantitative Analysis, Version B.07.00 /Build 7.0.457.0, 2008).

## Results and discussion

### Analytical method

The method applied in this study was developed based on a previous study with some modifications (Yamashita et al. 2004). As demonstrated in Table 1, the analytical method proved to be well-suited for this study with respect to linearity range, compound-specific linearity (*R*^2^) equal to or higher than 0.99 for all target PFASs, sensitivity (instrument and method limits of detection (ILODs and MDL) in the lower ng/L range for all PFASs, and extraction efficiency (relative recovery (%R) higher than 74% for all compounds except PFBA (17%)). Percent relative standard deviations (% RSD) were also found to be acceptable for all PFASs except for PFBA (33.8%) with all PFASs at or below 10% indicating good repeatability. Therefore, PFBA was excluded from quantification. These recoveries with this repeatability indicate that the negative effects of matrix suppression and SPE loss on quantification have been compensated by the use of isotope dilution. These results confirmed that all target PFASs (except PFBA) can be quantified using the here applied SPE extraction method (HLB based) and HPL-ESI-QqQ quantification. It is important to mention that our QC results confirmed the conclusions by Taniyasu et al. (2005): these authors tested the recoveries of PFASs extracted with SPE using HLB cartridges. They reported recoveries of > 80%, except for short-chain carboxylic acids such as PFHxA, PFPeA, and PFBA, for which recoveries were found less than 30% (Taniyasu et al. 2005).

**Table 1:** Instrumental limit of detection (ILOD), limit of quantification (LOQ), method detection limit (MDL) and relative recovery (%) of 50 ng/L spiked concentration in 1 L seawater. For abbreviation and names, see Table S1

Compound	ILOD (ng/mL)	LOQ (ng/L)	MDL (ng/L)	Rel. Recovery ± RSTD (%) (*n* = 4)
PFBA	0.074	0.11	4.1	17.4 ± 34
PFHxA	0.12	0.20	1.2	91.1 ± 1.8
PFHpA	0.15	0.85	1.1	81.1 ± 2.6
PFOA	0.044	0.27	0.42	96.0 ± 1.5
PFNA	0.10	0.17	3.0	93.5 ± 3.8
PFDA	0.044	0.074	0.18	87.0 ± 2.2
PFUnDA	0.01	0.13	0.094	93.3 ± 3.0
PFDoDA	0.054	0.084	0.063	86.1 ± 8.7
PFBS	0.054	0.084	0.68	74.3 ± 4.9
PFHxS	0.21	0.55	0.55	91.6 ± 3.2
L-PFOS	0.064	0.36	1.5	98.1 ± 5.7
6:2 FTS	0.49	0.81	0.94	117 ± 9.0
FOSA	0.014	0.024	0.044	93.0 ± 6.6

Relative recoveries of the isotope-labeled internal standards from seawater are shown in Table 2. Although only five ISTDs showed recoveries greater than 60%, the recoveries of all ISTDs were considered acceptable since these calculated absolute recoveries do not account for the losses during the sample preparation and matrix effects. For detailed information on chromatographic performance, detection, and quantification, please refer to the comprehensive descriptions in the supplementary materials section.

**Table 2:** Relative recovery (%) of spiked ISTD concentration from 25 ng/L in 1-L seawater

ISTD	Relative recovery ± RSTD (%)
[^13^C_4_]-PFBA	10.7 ± 30.0
[^13^C_5_]-PFHxA	64 ± 4.0
[^13^C_4_]-PFHpA	85 ± 4.0
[^13^C_4_]-PFOA	81 ± 1.0
[^13^C_5_]-PFNA	60.0 ± 3.2
[^13^C_2_]-PFDA	29 ± 15
[^13^C_2_]-PFUnDA	14 ± 15
[^13^C_2_]-PFDoDA	9.8 ± 2.2
[^18^O_2_]-PFHxS	83 ± 4.0
[^13^C_4_]-PFOS	22 ± 11
[^2^H_3_]-MeFOSA	30.0 ± 18

### Occurrence of PFASs in the eastern Red Sea coastal water

**Fig. 2:**
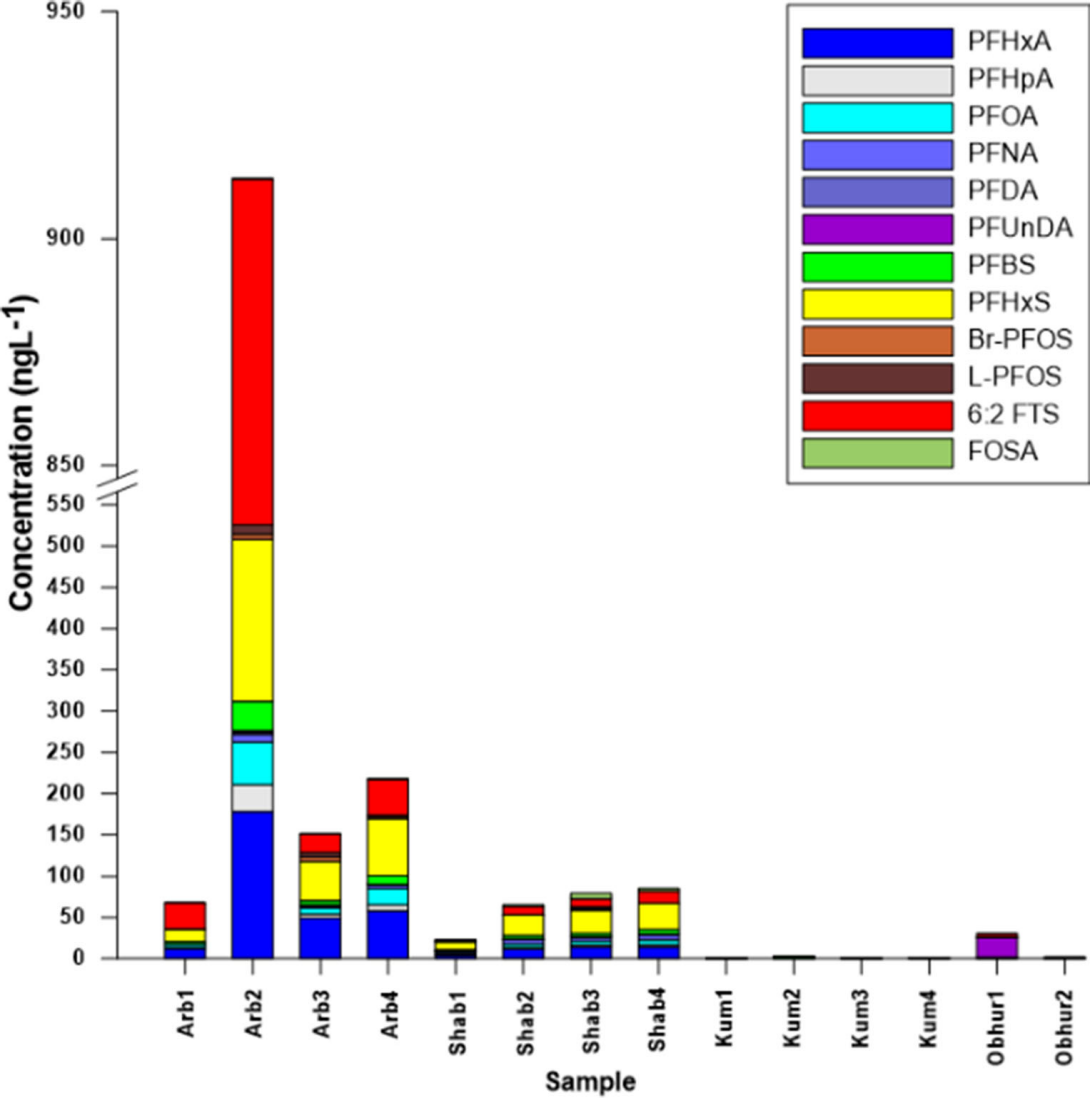
∑_12_PFASs levels (ng/L) in surface seawater samples from Al-Arbaeen (Arb 1–4), Al-Shabab (Shab 1–4), and Al-Khumrah (Kum 1–4) for location characteristics, see Fig. 1 and Ali et al. (2017)

For the first time, background surface seawater (Obhur) and contaminated seawater from effluent-impacted locations near Jeddah’s urban centers (Al-Khumrah, Al-Shabab, and Al-Arbaeen) were analyzed and quantified for selected PFASs (Fig. 1; Figs. S3-S31 and Tables S5 in the SM). Out of the 12 target PFASs, 11 PFASs were detected at concentrations exceeding the LOQs in one or more samples, as shown in Table 3 and S5 and Fig. 2. The sum of PFASs (∑_12_PFASs) in the Saudi coastal water of the Red Sea ranged from <LOQ ng L^−1^ to 956 ngL^−1^ (Fig. 2 and Table S5). ∑_12_PFASs in Al-Arbaeen samples were found among the highest contaminated among all analyzed samples (69.4–956 ngL^−1^) followed by ∑_12_PFASs in the Al-Shabab samples (4.4–89 ngL^−1^). This was expected, as these waters receive large amounts of effluents from nearby STPs (see “Sampling area” section) as well as firefighting stations. Recently, STPs have been identified as significant sources for organic contaminants of emerging concern (CECs) in these locations (Al-Lihaibi et al. 2019; Ali et al. 2017; Ali et al. 2018). Variations in effluent load and hydrological differences in the water exchange of the lagoons with the open sea explain the difference in ∑_12_ PFASs levels found in water samples of the two lagoons. ∑_12_ PFASs in samples collected from Al-Khumrah and Obhur were relatively low (<LOQ − 10.7 ngL^−1^). C_6_–C_9_ PFCAs were the dominant PFCAs in all samples analyzed, and PFOA was the dominant PFAA (detection frequency of 96%) ranging from nd to 66 ng L^-1^. C_4_ and C_6_ PFSAs were the dominant PFSAs in samples analyzed having detection frequencies of 60 and 57%, respectively, and maximum concentrations of 50.8 and 245 ng L^−1^ respectively. 6:2 fluorotelomer sulfonate (6:2 FTS) was detected in 76% of the samples with a maximum concentration of 325 ng L^−1^.

**Table 3:** Levels (range (median) in ng/L) of PFAS from four locations in the eastern coastal waters of the Red Sea near Jeddah (KSA)

Compound	Al-Arbaeen (*n* = 8)	Al-Shabab (*n* = 7)	Al-Khumrah (*n* = 9)	Obhur (*n* = 4)
PFHxA	0.223 to > 198 (57.8)	1.22–15.8 (10.5)	Nd	Nd–0.743 (Nd)
PFHpA	<LOQ−54.1 (7.83)	0.924–3.03 (1.73)	Nd–0.334 (Nd)	Nd–0.934 (0.453)
PFOA	0.345–66.0 (10.9)	0.844–7.73 (3.42)	Nd–1.045 (0.776)	Nd–1.53 (1.11)
PFNA	<LOQ–15.6 (3.15)	<LOQ–5.93 (3.83)	Nd–0.545 (Nd)	Nd
PFDA	Nd–5.63 (Nd)	Nd−1.52 (0.545)	Nd	Nd
PFUnDA	Nd–5.66 (Nd)	Nd–0.223 (Nd)	Nd	Nd–23.8 (11.9)
PFBS	Nd–50.85 (8.8)	Nd–5.52 (3.62)	Nd–4.93 (Nd)	Nd
PFHxS	Nd–245 (68.9)	Nd–37.3 (19.9)	Nd	Nd–0.734 (Nd)
Br-PFOS	<LOQ–12.94 (0.334)	Nd–4.44 (Nd)	Nd	Nd
L-PFOS	<LOQ −21.6 (0.434)	Nd–4.77 (Nd)	Nd	Nd
6:2 FTS	0.135 to > 450 (51.4)	0.823–14.4 (7.3)	Nd–0.345 (Nd)	Nd–8.62 (0.634)
FOSA	Nd–0.843 (0.482)	Nd to > 12.54 (2.64)	Nd	Nd

**Fig. 3:**
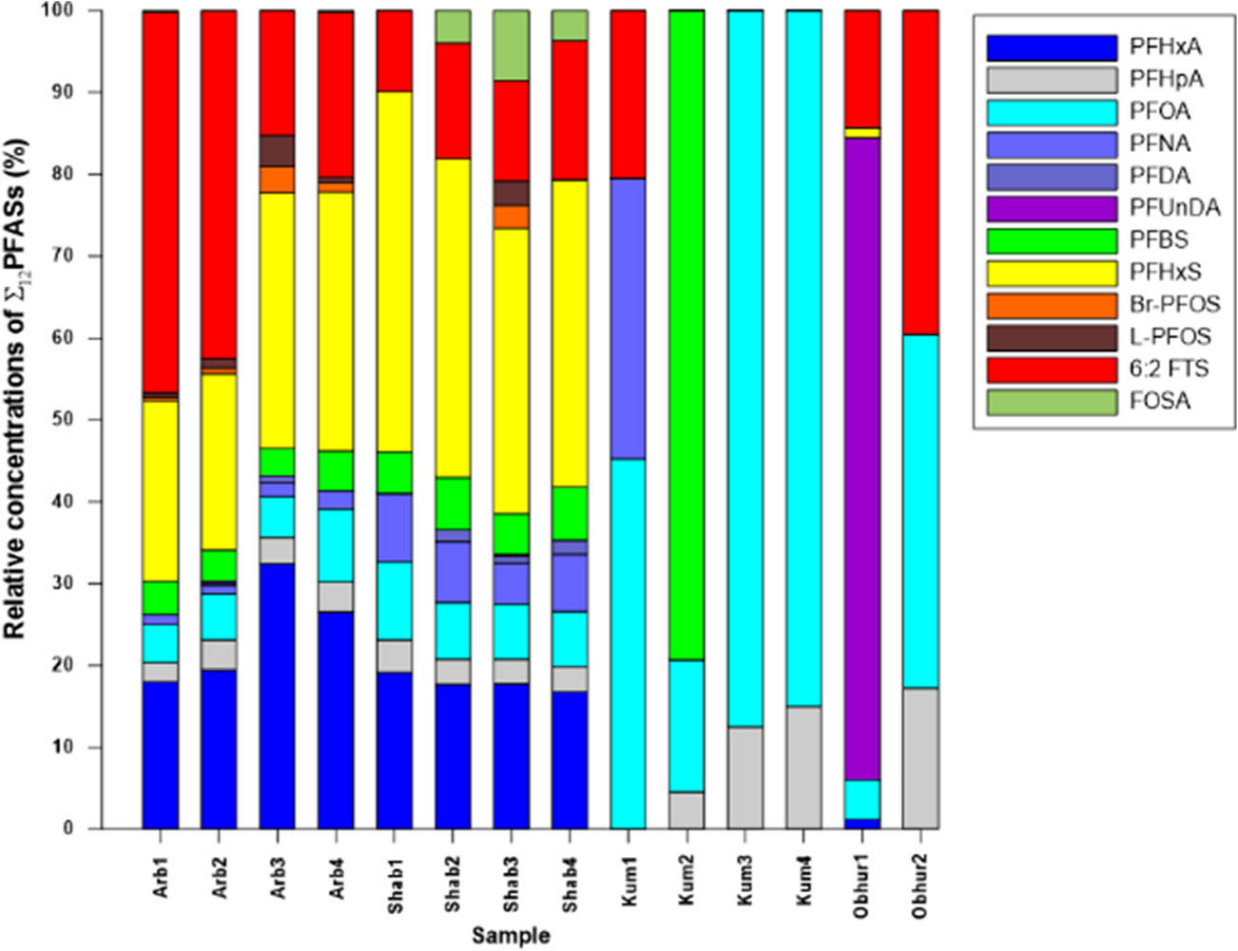
Profile of relative abundance for individual PFASs in relation to ∑_12_PFASs in the eastern waters of the Red Sea near Jeddah (KSA)

As depicted in Figs. 3, patterns for the relative abundance of PFASs varied among locations, which may indicate different potential sources of these compounds. 6:2 FTS, PFHxA, and PFHxS were the most prevalent compounds in samples from Al-Arbaeen (Arb1–4) and Al-Shabab (Shab 1–4), accounting for 64–84% of ∑_12_ PFAS. As mentioned before (“Sampling area” section), these two locations receive continuous and untreated effluent from Jeddah city, including domestic sewage and potential effluents from the nearby firefighting stations. It is noteworthy that samples Arb2, Arb4, and Shab4 displaying relatively higher concentrations of ∑_12_ PFAS (Fig. 2) were collected from sites which are closer to the effluents (domestic and firefighting station sewage) at Al-Arbaeen and Al-Shabab lagoons (see Fig. 1). This indicates that effluents are potential source of these compounds. However, wastewater effluent is considered a major PFAS source (Campo et al. 2014; Cerveny et al. 2018; Dauchy et al. 2017). Therefore, the wastewater effluent is likely the major source for elevated PFAS levels in these lagoons.

Only PFOA and PFHpA were detected in the Al-Khumrah water samples at mean concentrations of 0.3 and 0.6 ng L^−1^, respectively. With regard to the reference site, PFOA, PFHpA, and 6:2 FTS are the predominant PFASs at the Obhur background location with concentrations of 1.1, 0.4, and 2.5 ng L^−1^, respectively. The Al-Khumrah site receives a tertiary treated sewage discharge from Al Khumrah STP; however, the observed PFASs levels are relatively low (0.6–6.7 ng/L) compared with their corresponding levels in Al-Arbaeen and Al-Shabab. Overall, PFOA was the most ubiquitous compound in all seawater (detection frequency (df%) of 96%) followed by 6:2 FTS (df% = 76%), PFHpA (df% = 75%), PFHxA (df% = 57%), PFHxS, (df% = 57%), PFBS (df% = 57%), PFOS, PFNA (df% = 57%), and finally FOSA(df% = 28%). The here-reported PFAS levels are found to be comparable with PFAS profiles reported in other similar international studies as summarized in Table S6. In comparison, PFOA was the most ubiquitous compound in Mediterranean seawater samples collected from STP-influenced sampling locations (76%) followed by PFNA (69%), PFOS (62%), PFHxS (34%), and finally PFBS (10%) (Sánchez-Avila et al. 2010). Compared with our results, PFHxA, PFHpA, PFOA, PFHxS, and PFOS were also the most abundant PFASs in seawater samples collected from the open Western Mediterranean Sea, where the sum of PFAS concentrations in surface seawater ranged from 0.25 to 0.52 ng/L (Brumovský et al. 2016). PFOA was the predominant PFASs, followed by PFOS, in water samples collected from coastal areas in Japan (Yamashita et al. 2005). Another Mediterranean study also confirmed PFOS and PFOA as the two major PFASs in Catalonian coastal waters followed by PFHxS, PFNA, and PFBS (Sánchez-Avila et al. 2010). We assume, thus, that the PFHxA, PFHxS, and 6:2 FTS-dominated profiles found here indicate largely application of industrial productions in Saudi Arabia mainly in form of ingredients in consumer products or in AFFF for large-scale fire protection. The relatively high concentration of PFASs in the Al-Arbaeen and Al-Shabab lagoon indicates that the receiving secondary treated sewage in local STPs is likely the predominant source for this location, as confirmed already for other compound groups earlier (Ali et al. 2017).

Like for other organic pollutants, PFAS levels in surface seawater are controlled by their input from the sources and their removal processes such as advection, dilution, diffusion, degradation, and sedimentation. As shown here, STPs represent a major source for PFAS contamination in the Red Sea coastal water at Jeddah. Adhesion to suspended particles and sedimentation is a potential removal process in Jeddah coastal waters. PFAS level reduction by the export of marine aerosols (McMurdo et al. 2008) is also a feasible removal process in the coastal Red Sea.

**Fig. 4:**
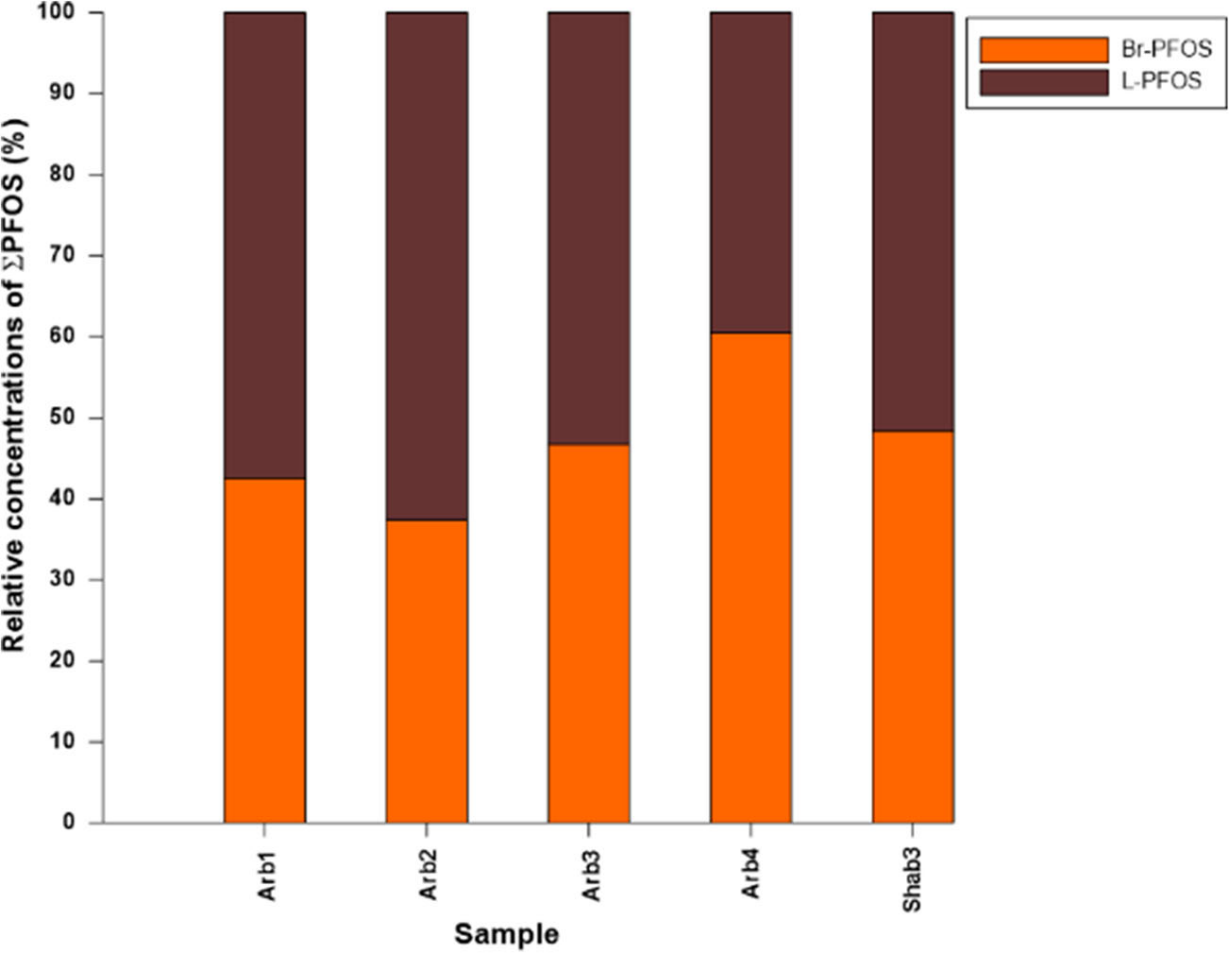
Branched (Br-PFOS) and linear (L-PFOS) composition profile (relative distribution) in selected contaminated water samples (Arb and Shab) from Jeddah coastal water

PFOS is found both as linear *n*-alkane and branched isomer in environmental samples. The PFOS isomer profile for selected sites is shown in Fig. 4. For the performed study, the same instrumental sensitivity and linearity as determined for the linear PFOS were assumed (therefore, potential uncertainties with respect to calculated concentration values must be considered when interpreting these data). The proportion of branched PFOS ranged from 37 to 60%. The increased proportion of branched isomers compared to the technical mixture indicates enrichment of branched isomers in the Red Sea surface coastal water. The physicochemical properties of the linear and branched PFOS vary slightly, which may lead to differences in their adsorption properties onto solid surfaces. PFASs branched isomers have been predicted to be more hydrophilic compared with the linear PFOS (Shoeib et al. 2011). This explains the observed enrichment of branched isomers in the Red Sea coastal surface water, as linear PFOS may be adsorbed more efficiently on suspended particles and consequently removed to sediments on the ocean floor.

Furthermore, the ratio of linear and branched isomers can be influenced by preferential elimination of branched isomers in biochemical processes as also shown for human physiology (Greaves and Letcher 2013). Therefore, PFOS in the STPs originally excreted by humans are expected to have enriched branched isomers. Furthermore, branched precursors have been shown to transform in biota more rapidly than the linear isomer precursors (Benskin et al. 2009), which could also explain this observed enrichment. It is important to note that the levels of PFOS in the coastal water of the Red Sea at Jeddah are relatively low compared with 6:2 FTS. It has been proposed earlier that sedimentation of suspended particles can act as a sink for certain PFASs such as PFOS or longer-chain PFASs (Higgins et al. 2006). This may cause the here-observed decrease in PFOS concentration. It is worth mentioning that the reported levels of PFASs in this study represent the dissolved PFASs since all seawater samples were filtered before the SPE extraction. Additionally, 6:2 FTS is an alternative to PFOS in several industrial products such as metal plating and fluoropolymers (Urtiaga et al. 2018).

Al-Arbaeen and the Al-Shabab locations were found to be contaminated by PFASs (Fig. 3) with PFAS levels up to 956 ng/L (Al-Arbaeen) and 89 ng/L (Al-Shabab), respectively. Especially for the Arb2, Arb 4, Shab2, and Shab4 samples (collected close to the effluents), unusual PFAS patterns were identified indicating a predominant primary contamination source. The sample-specific patterns are remarkably similar at both locations, though the sum PFAS concentration is roughly 8 times higher at Al-Arbaeen (Table 3 and Fig. 3). At both locations, 6:2 FTS, PFHxA, and PFHxS stand for 64–84% of ∑_12_PFAS (Fig. 3). These three PFASs are frequently reported at elevated ratios at AFFF-impacted sites (Barzen-Hanson et al. 2017; D’Agostino and Mabury 2017; Hoisaeter et al. 2019; Houtz et al. 2018; Mejia-Avendano et al. 2017; Milley et al. 2018; Munoz et al. 2017c; Xiao et al. 2017).

Two firefighting stations serving the area around Al-Arbaeen and Al-Shabab were identified ca. 400 m from the lagoons (Fig. 1). Furthermore, STPs are located between the lagoons and the firefighting stations (ca. 150 m from each lagoon), where potential aqueous wastes after regular firefighting training exercises may be collected and transported further as effluent water after treatment into the lagoons (Fig. 1).

According to the available official information on the wastewater treatment system, only sedimentation and secondary treatment (as shown in the overview Fig. 1) are employed in the plants nearby Al-Arbaeen and Al-Shabab. Thus, PFAS contamination of the resulting effluent waters must be considered very likely. Furthermore, the vicinity of the fire training facility close to the sample locations is an indication of a potential primary PFAS emission source. Thus, further investigation on the use and application of AFFF during the regular training programs of the firefighters stationed here would enable further in-depth PFAS source elucidation at the location. Unlike Al-Arbaeen and Al-Shabab (semi-enclosed lagoons (see Fig. 1), which receive secondary treated sewage from STPs, the low levels in the water samples from Al-Khumrah site are likely indicative of efficient removal of PFASs during tertiary treatment applied in the Al-Khumrah STP. Additionally, higher dilution in the receiving effluent waters resulted in sufficient water exchange with the open sea in Al-Khumrah compared with the semi-closed lagoons. Furthermore, the difference in the composition of sewage (domestic waste and firefighting stations wastes in the Al-Arbaeen and Al-Shabab and industrial wastes in Al-Khumrah) cannot be excluded as an additional factor contributing the low PFAS levels observed.

In contrast, Obhur is considered rural/ sub-urban background-level location for PFASs’ contaminants with likely minor secondary, diffusive sources. The relative vicinity of the Obhur station to Al-Arbaeen (Fig. 1), however, likely contributes to potential contamination from “high concentration” emission events as indicated in sample Obhur 1 and 2 (with predominant levels of 6:2 FTS and up to 10 ng/L ∑PFASs). However, in most cases, the ∑PFAS concentrations are found below 4 ng/L, confirming the general relatively low PFAS concentrations along the Jeddah coast.

The relative PFAS distribution profiles in the 28 seawater samples analyzed and quantified from four representative coastal locations from the Jeddah coast (Eastern Saudi Red Sea) revealed that predominant local sources are causing continuous PFAS pollution in the polluted waters of Al-Arbaeen and Al-Shabab lagoons (Fig. 2). For these lagoons, mainly 6:2 FTS, PFHxS, and PFHxA are the predominant constituents of our PFAS target compound list (Fig. 3). The background locations (Obhur), however, expressed large variability in the sample-specific PFAS patterns and just confirmed the contribution of varying diffusive sources with an occasional predominance of nearby contaminated waters.

The here-identified characteristic PFAS level and pattern profile confirmed the predominant contribution of a primary source to the PFAS effluent from an STP applying secondary treatment processing. High PFAS levels were identified at two sampling locations, Al-Arbaeen and Al-Shabab in the close vicinity of a secondary treatment STPs. Three PFASs are identified as the dominating compounds (64–84% of ∑_11_PFAS) in the water samples (6:2 FTS, PFHxS, and PFHxA). The identified profile suggests a release of AFFF to the treatment plant. Most probably, AFFF was or is regularly used during training events and thereafter enters the STPs and is released through the effluent. However, the here-proposed source identification is only preliminary and further confirmation would be needed.

## Conclusion

In summary, a PFAS quantification method was adjusted and validated for the trace quantification of 12 PFASs in seawater samples from contaminated locations near Jeddah (KSA) in the eastern Red Sea. PFASs’ concentrations were determined in water samples from four locations with different expected PFAS source contributions. Relatively high concentrations of PFHxA, PFHxS, and 6:2 FTS were found for the Al-Arbaeen and Al-Shabab locations close to local STPs. The possible source is most likely local industries or firefighting training facilities close to the treatment plants. The relative homogeneous PFAS distribution in the lagoons’ seawater samples indicates continuous, high-level discharge. Thus, the discharges of only partially treated sewage from Jeddah city is considered the major source of these contaminants. There is an urgent need to consider reductions in sewage discharge into Al-Arbaeen and Al-Shabab lagoons to protect the coastal environment. Additionally, a comprehensive ecological study taking into consideration the assessment of PFASs in sediment biota and drinking waters in Jeddah should be undertaken.

## Electronic supplementary material

ESM 1 (DOCX 1159 kb)
