# Supplementary material for: Per- and polyfluoroalkyl substances (PFASs) in contaminated coastal marine waters of the Saudi Arabian Red Sea: a baseline study
Source: Environ Sci Pollut Res Int. 2020 Sep 7;28(3):2791–803. doi: 10.1007/s11356-020-09897-5 (PMC7788036; doi:10.1007/s11356-020-09897-5)
Supplement: Supplementary file 1 — (DOCX 1159 kb) [file 11356_2020_9897_MOESM1_ESM.docx]

**Per-and polyfluoroalkyl substances (PFASs) in Contaminated Coastal Marine Waters of the Saudi Arabian Red Sea: A baseline study**

# Aasim M. Ali^1*^, Christopher P. Higgins^2^, Walied M. Alarif^3^, Sultan S. Al- Lihaibi^3^, Mohammed Ghandourah^3^, Roland Kallenborn^1,4^

## ^1^Faculty of Chemistry, Biotechnology and Food Sciences, Norwegian University of Life Sciences, Christian M. Falsen veg 1, NO-1432, ÅS, Norways

^2^Department of Civil & Environmental Engineering, Colorado School of Mines, 1500 Illinois Street, Golden, Colorado 80401, United States

## ^3^ Department of Marine Chemistry, Faculty of Marine Sciences, King Abdulaziz University, PO. Box 80207, Jeddah 21589, Saudi Arabia

^4^ Arctic Technology Department (AT), University Centre in Svalbard (UNIS), P.O. Box 156, Longyearbyen. Svalbard, Norway.

Corresponding Author: Aasim M. Al: [aasimali@nmbu.no](mailto:aasimali@nmbu.no), Tel.: [+4767232579](tel:+4767232579)

Table S1 List of target PFASs for quantitative analysis

| **Analyte Name** | **Acronym** | **CAS #** | **Formula** | **Structure** | **LogP** | **LogD (7.4)** |
| --- | --- | --- | --- | --- | --- | --- |
| **PFCAs** | | | | | | |
| Perfluorobutanoic acid | PFBA | 375-22-4 | F(CF_2_)_3_COOH |  | 3.94 | -1.13 |
| Perfluoropentanoic acid | PFPeA | 2706-90-3 | F(CF_2_)_4_COOH |  | 5.29 | -0.34 |
| Perfluorohexanoic acid | PFHxA | 307-24-4 | F(CF_2_)_5_COOH |  | 5.97 | 0.15 |
| Perfluoroheptanoic acid | PFHpA | 375-85-9 | F(CF_2_)_6_COOH |  | 6.86 | 1.11 |
| Perfluorooctanoic acid | PFOA | 335-67-1 | F(CF_2_)_7_COOH |  | 7.75 | 1.82 |
| Perfluorononanoic acid | PFNA | 375-95-1 | F(CF_2_)_8_COOH |  | 8.64 | 2.84 |

Table S2 continue

| **Analyte Name** | **Acronym** | **CAS #** | **Formula** | **Structure** | **LogP** | **LogD (7.4)** |
| --- | --- | --- | --- | --- | --- | --- |
| **PFCAs** | | | | | | |
| Perfluorodecanoic acid | PFDA | 335-76-2 | F(CF_2_)_9_COOH |  | 9.53 | 3.62 |
| Perfluoroundecanoic acid | PFUnDA | 2058-94-8 | F(CF_2_)_10_COOH |  | 10.42 | 4.23 |
| Perfluorododecanoic acid | PFDoDA | 307-55-1 | F(CF_2_)_11_COOH |  | 11.31 | 4.58 |
| Perfluorotridecanoic acid | PFTriDA | 72629-94-8 | F(CF_2_)_12_COOH |  | 12.19 | 4.97 |
| **PFSAs** | | | | | | |
| Perfluorobutane sulfonic acid | PFBS | 375-73-5 | F(CF_2_)_4_SO_3_H |  | 3.68 | -1.56 |
| Perfluorohexane sulfonic acid | PFHxS | 355-46-4 | F(CF_2_)_6_SO_3_H |  | 5.25 | -0.54 |
| Perfluorooctane sulfonic acid | PFOS | 1763-23-1 | F(CF_2_)_8_SO_3_H |  | 7.03 | 0.66 |
| **FTSAs** |  |  |  |  |  |  |
| 6:2 Fluorotelomer sulfonic acid | 6:2 FTS | 27619-97-2 | F(CF_2_)_6_(CH_2_)_2_SO_3_H |  | 3.47 | -1.00 |


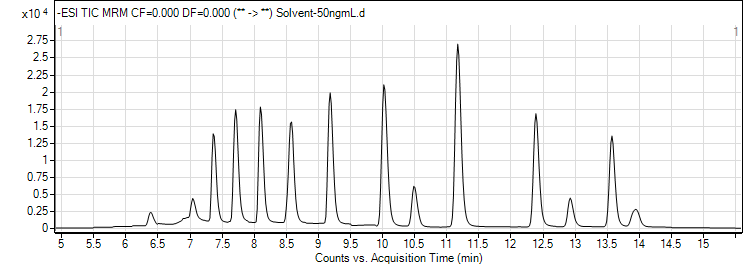


*Figure S1 Total ion chromatogram of all compounds at 50pg/µL*


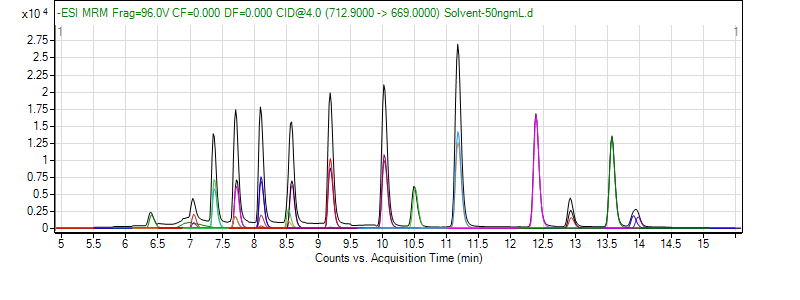


Figure S2 Extracted and Overlaid MRM of all compounds at 50pg/µL

Table S3 Agilent 6460 ionization source (JetStream) parameters

| **Parameter** | **Value** |
| --- | --- |
| Gas Temp (°C) | 300 |
| Gas Flow (l/min) | 5 |
| Nebulizer (psi) | 25 |
| SheathGasHeater | 400 |
| SheathGasFlow | 8 |
| Capillary (V) | -2500 |
| VCharging | 500 |

Table S4 MRM transitions of target PFAS compounds

| Acronym | ISTD used | Retention time  [min] | Precursor ion  (m/z) | Product ion 1 (Quantifier) (m/z) | Product ion 2  (Qualifier) (m/z) | CE1  (V) | CE2  (V) | Fragmentor  (V) |
| --- | --- | --- | --- | --- | --- | --- | --- | --- |
| PFBA | [^13^C_4_]-PFOA | 6.4 | 213 | 169 |  | 1 |  | 61 |
| PFHxA | [^13^C_5_]-PFHxA | 7.3 | 313 | 269 | 119 | 0 | 19 | 66 |
| PFHpA | [^13^C_4_]-PFHpA | 7.6 | 363 | 319 | 168.9 | 0 | 10 | 71 |
| PFOA | [^13^C_4_]-PFOA | 8.0 | 413 | 369 | 169 | 0 | 12 | 76 |
| PFNA | [^13^C_5_]-PFNA | 8.4 | 463 | 419 | 219 | 4 | 4 | 86 |
| PFDA | [^13^C_2_]-PFDA | 9.1 | 513 | 469 | 169 | 4 | 4 | 86 |
| PFUnDA | [^13^C_5_]-PFNA | 10 | 563 | 519 | 169 | 4 | 10 | 86 |
| PFDoDA | [^13^C_5_]-PFNA | 7 | 613 | 569 |  | 4 |  | 96 |
| PFBS | [^18^O_2_]-PFHxS | 7 | 299 | 99 | 80 | 25 | 35 | 121 |
| PFHxS | [^18^O_2_]-PFHxS | 7 | 398.9 | 99 | 80 | 45 | 45 | 151 |
| Br-PFOS | [^18^O_2_]-PFHxS | 8.3 | 499 | 99 | 80 | 61 | 61 | 200 |
| L-PFOS | [^18^O_2_]-PFHxS | 8.4 | 499 | 99 | 80 | 61 | 61 | 200 |
| 6:2 FTS | [^18^O_2_]-PFHxS | 8 | 427 | 407 | 81 | 15 | 15 | 145 |
| FOSA | [^2^H_3_]-MeFOSA | 10.5 | 497.9 | 78 |  | 33 |  | 141 |

Table S5 MRM transitions of internal standards

| Acronym | Retention time [min] | Precursor ion  (m/z) | Product ion 1 (Quantifier) (m/z) | CE  (V) | Fragmentor |
| --- | --- | --- | --- | --- | --- |
| [^13^C_8_]-PFOA | 8.34 | 421 | 376 | 0 | 76 |
| [^13^C_4_]-PFBA | 6.58 | 217 | 172 | 1 | 61 |
| [^13^C_5_]-PFHxA | 7.66 | 318 | 273 | 0 | 66 |
| [^13^C_4_]-PFHpA | 7.99 | 367 | 322 | 0 | 66 |
| [^13^C_4_]-PFOA | 8.35 | 417 | 372.1 | 0 | 76 |
| [^13^C_5_]-PFNA | 8.79 | 468 | 423 | 4 | 76 |
| [^13^C_2_]-PFDA | 9.34 | 515 | 470 | 4 | 86 |
| [^13^C_2_]-PFUnDA | 9.94 | 565 | 520 | 4 | 96 |
| [^13^C_2_]-PFDoDA | 10.86 | 615 | 570 | 4 | 96 |
| [^18^O_2_]-PFHxS | 7.97 | 403 | 84 | 49 | 146 |
| [^13^C_4_]-PFOS | 8.74 | 503 | 80 | 61 | 180 |
| [^2^H_3_]-MeFOSA | 9.34 | 515 | 169 | 25 | 136 |

**Table S6 Levels of PFAS in Selected Sites in the Eastern Coastal Waters of the Red Sea**

| **Sample** | **PFHxA** | **PFHpA** | **PFOA** | **PFNA** | **PFDA** | **PFBS** | **PFHxS** | **PFOS** | **6:2 FTS** | **FOSA** | **∑_10_PFASs** |
| --- | --- | --- | --- | --- | --- | --- | --- | --- | --- | --- | --- |
| Arb1-A | 0.026 | <LOQ | <LOQ | <LOQ | <LOQ | <LOQ | <LOQ | <LOQ | <LOQ | <LOQ | 3.20 |
| Arb1-B | 24.5 | 3.09 | 6.26 | 1.74 | Nd | 5.51 | 29.89 | 1.36 | 63.0 | 0.243 | 135 |
| Arb2-A | 157 | 12.5 | 36.8 | 2.24 | Nd | 50.8 | 245 | 0.083 | 45.0 | 0.482 | 956 |
| Arb2-B | 198 | 54.1 | 66.0 | 15.6 | 5.62 | 18.1 | 147 | 34.5 | 325 | Nd | 865 |
| Arb3-A | 84.7 | 9.86 | 11.4 | 5.04 | 2.56 | 10.11 | 94.4 | 21.4 | 43.5 | Nd | 283 |
| Arb3-B | 13.4 | <LOQ | 3.67 | <LOQ | <LOQ | <LOQ | <LOQ | <LOQ | 2.56 | Nd | 21.7 |
| Arb4-A | 62.2 | 5.79 | 10.3 | 4.02 | Nd | 13.4 | 73.1 | <LOQ | 28.4 | 0.843 | 198 |
| Arb4-B | 53.3 | 10.1 | 28.6 | 5.85 | Nd | 7.57 | 64.81 | 7.83 | 59.4 | Nd | 237 |
| Shab1 | 1.21 | 0.415 | 0.826 | <LOQ | Nd | <LOQ | <LOQ | <LOQ | 0.796 | Nd | 4.38 |
| Shab2-A | 7.55 | 1.42 | 3.53 | 3.84 | Nd | 2.29 | 20.1 | <LOQ | 3.74 | Nd | 42.9 |
| Shab2-B | 8.93 | 1.31 | 3.38 | 3.80 | 0.484 | 2.74 | 19.1 | <LOQ | 4.10 | 2.00 | 46.2 |
| Shab3-A | 14.3 | 2.63 | 5.72 | 5.95 | 1.48 | 5.55 | 32.0 | <LOQ | 14.4 | 3.18 | 85.5 |
| Shab3-B | 15.8 | 2.99 | 7.73 | 7.02 | 1.42 | 3.62 | 37.3 | <LOQ | 12.1 | 1.15 | 89.5 |
| Shab4-A | 12.4 | 1.66 | 3.01 | 0.92 | Nd | 4.29 | 18.1 | <LOQ | 7.32 | 12.5 | 60.5 |
| Shab4-B | 10.5 | 1.83 | 3.36 | 0.715 | 1.30 | 7.53 | 19.9 | <LOQ | 12.0 | Nd | 57.5 |
| Kum1-A | Nd | Nd | 0.664 | <LOQ | Nd | Nd | Nd | Nd | Nd | Nd | 0.834 |
| Kum1-B | Nd | Nd | Nd | Nd | Nd | Nd | Nd | Nd | <LOQ | Nd | 0.816 |
| Kum2-A | Nd | 0.285 | 0.993 | Nd | Nd | <LOQ | Nd | Nd | Nd | Nd | 1.35 |
| Kum2-B | Nd | <LOQ | Nd | Nd | Nd | <LOQ | Nd | Nd | Nd | Nd | <LOQ |
| Kum3-A | Nd | Nd | 0.592 | Nd | Nd | Nd | Nd | Nd | Nd | Nd | 0.592 |
| Kum3-B | Nd | 0.264 | 0.562 | Nd | Nd | Nd | Nd | Nd | Nd | Nd | 0.833 |
| Kum3-C | Nd | Nd | 0.672 | Nd | Nd | Nd | Nd | Nd | Nd | Nd | 0.674 |
| Kum4-A | Nd | Nd | 0.913 | Nd | Nd | Nd | Nd | Nd | Nd | Nd | 0.914 |
| Kum4-B | Nd | 0.304 | 0.774 | Nd | Nd | Nd | Nd | Nd | Nd | Nd | 1.07 |
| Obur1-A | Nd | Nd | 1.37 | Nd | Nd | Nd | <LOQ | Nd | Nd | Nd | 1.92 |
| Obur1-B | <LOQ | Nd | 1.56 | Nd | Nd | Nd | Nd | 9.12 | 8.67 | Nd | 19.5 |
| Obur2-A | Nd | 0.293 | 0.585 | Nd | Nd | Nd | Nd | Nd | 1.28 | Nd | 2.15 |
| Obur2-B | Nd | 0.273 | 0.824 | Nd | Nd | Nd | Nd | Nd | Nd | Nd | 1.09 |
| **D. freq %** | **57.0** | **75.0** | **96.4** | **57.1** | **28.6** | **60.7** | **57.1** | **57.1**67.9 | **76.0** | **28.6** | **-** |

Table S6 Concentrations (ng/L) of selected PFASs in coastal and open ocean water samples from international studies

| **Location** | **PFBS** | **PFOS** | **PFHxS** | **PFNA** | **PFOA** | **Reference** |
| --- | --- | --- | --- | --- | --- | --- |
| Tokyo Bay | - | 0.338-57.7 | 0.017-56.0 | 0.163-71.0 | 1.80-192 | (Yamashita et al. 2005) |
| The German Baltic Sea | <MDL -0.430 | <MDL -0.180 | <MDL-0.480 | <MDL-0.21 | 0.200-0.700 | (Joerss et al. 2019) |
| Surface seawater from China | 0.189–2.66 | <MDL | 0.181–2.66 | - | (0-061–0.471 | (Zheng et al. 2017) |
| Coastal area of China | - | 0.023-9.68 | <0.005-1.36 | 0.002-0.692 | 0.243-15.3 | (Yamashita et al. 2005) |
| Coastal area of Korea | - | 0.039-2.53 | <0.005-1.39 | 0.015-0.518 | 0.239-11.353 | (Yamashita et al. 2005) |
| Western Pacific Ocean | - | 0.054-0.078 | 0.002-0.003 | - | 0.136-0.142 | (Yamashita et al. 2005) |
| Central to Eastern Pacific Ocean (surface water) | - | 0.001-0.02 | 0.0001-0.0016 | 0.001-0.016 | 0.015-0.062 | (Yamashita et al. 2005) |
| Central to Eastern Pacific Ocean (deep water; 4000–4400 m) | - | 0.003-0.0034 | 0.0004-0.0006 | - | 0.045-0.062 | (Yamashita et al. 2005) |
| North Atlantic Ocean | - | 0.008-0.036 | 0.0041-0.0061 | 0.015.0.036 | 0.160-0.338 | (Yamashita et al. 2005) |
| Mid Atlantic Ocean | - | 0.037-0.073 | 0.0026-0.012 | - | 0.100-0.439 | (Yamashita et al. 2005) |
| NW Mediterranean Sea (in Catalonia) - effluent water from municipal WWTP | 0.355–2.03 | 2.79–72.1 | 5.80–25.3 | 1.87–14.1 | 3.47–61.9 | (Sánchez-Avila et al. 2010) |
| Svalbard, Norwegian Arctic seawater | 0.035 | 0.100 | 0.210 | <0.020 | <0.030 | (Skaar et al. 2019) |
| Jeddah coast, Red Sea, KSA | n.d.-50.00 | n.d.-340 | n.d.-245 | n.d.-15.0 | n.d.-66.2 | This study |

Table 7 List of the used instruments and software

| **Item** | **Specification** | **Producer** | **Supplier** |
| --- | --- | --- | --- |
| MS | 6460 series  triple quadrupole LC/MS | Agilent  Technologies, Santa  Clara, CA, USA | Matriks AS, Oslo,  Norway |
| HPLC | Agilent 1200 series with  auto sampler, binary pump and column oven | Agilent  Technologies, Santa  Clara, CA, USA | Matriks AS, Oslo,  Norway |
| Software | MassHunter, Quantitative analysis for  QQQ, Versjon B.07.00/Build 7.0.457.0 | Agilent  Technologies, Santa  Clara, CA, USA | Matriks AS, Oslo,  Norway |
| Software | MassHunter, Qualititative analysis for  QQQ, Versjon B.06.00/Build 6.0.633.10 | Agilent  Technologies, Santa  Clara, CA, USA | Matriks AS, Oslo,  Norway |
| Evaporator | Reacti-Vap III™ Evaporator | Thermo Scientific,  Waltham, MA, USA | VWR  International AS,  Oslo, Norge |
| Vortex | MS 3 basic | IKA-Werke GmbH & Co, KG.  Wilmington, N.C, USA |  |


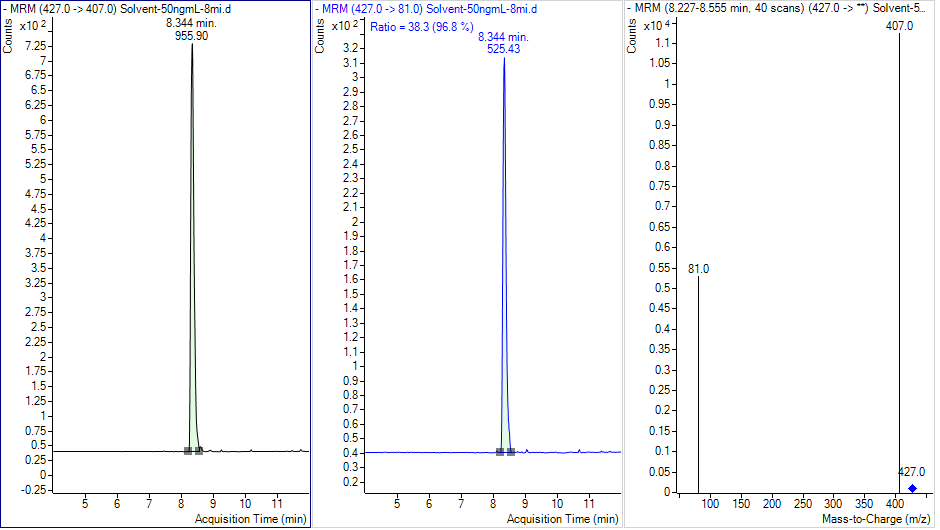


Figure S3 6:2 FTSA standard


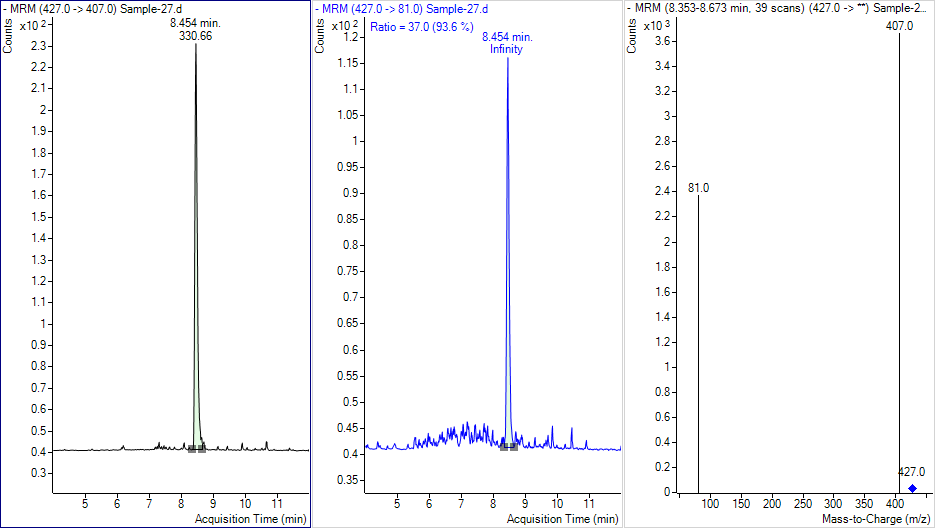


Figure S4 6:2 FTSA detected in sample


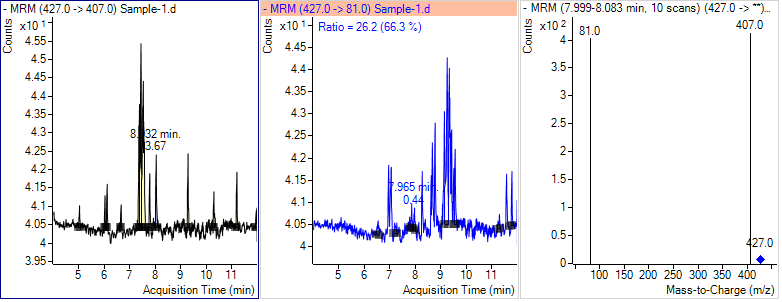


Figure S5 6:2 FTSA detected in field blank sample


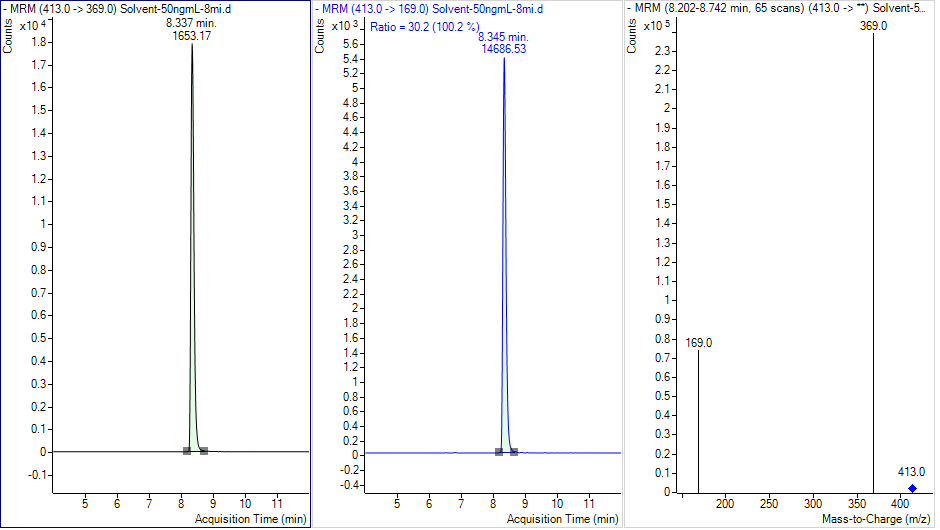


Figure S6 FOSA standard


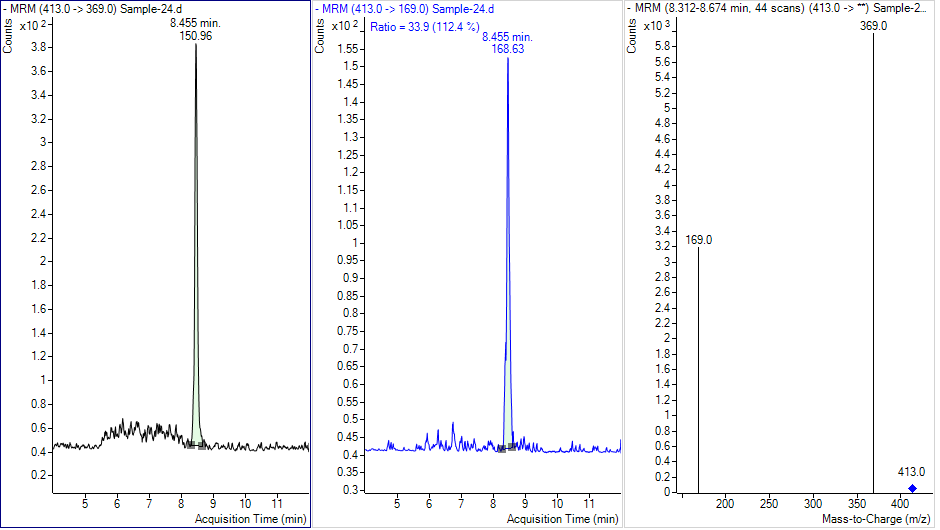


Figure S7 FOSA in Sample


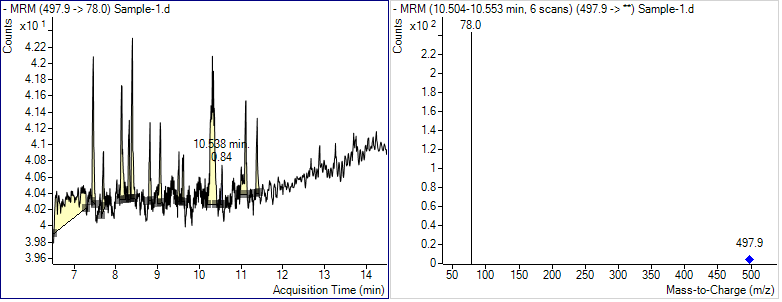


Figure S8 FOSA in blank sample


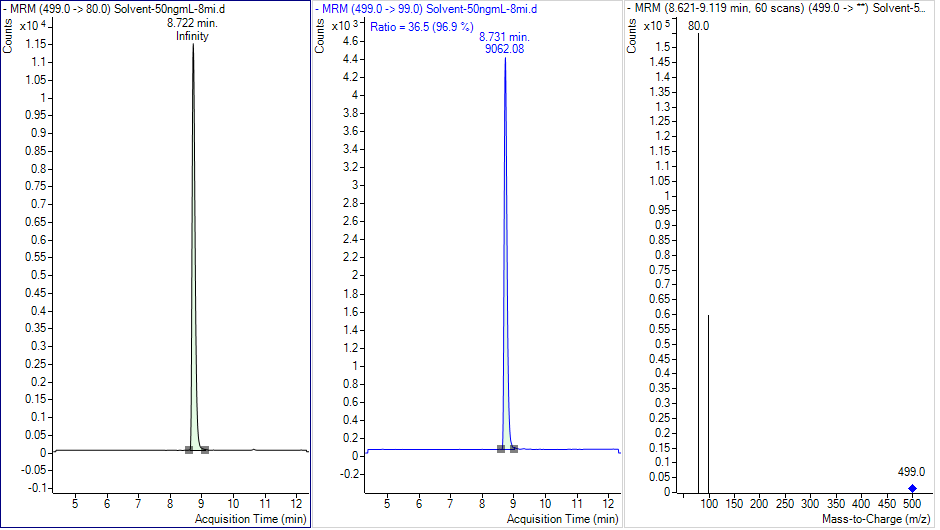


Figure S9 L-PFOS standard


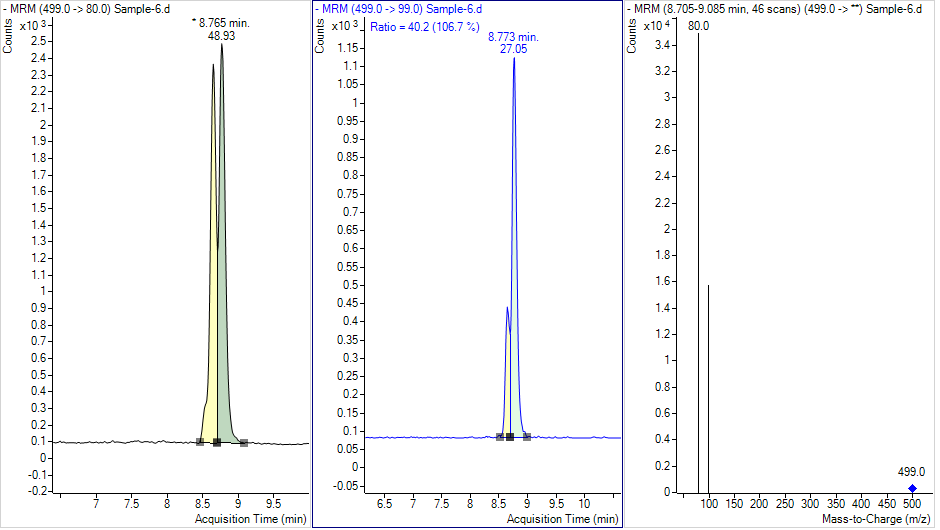


Figure S10 L-PFOS in sample


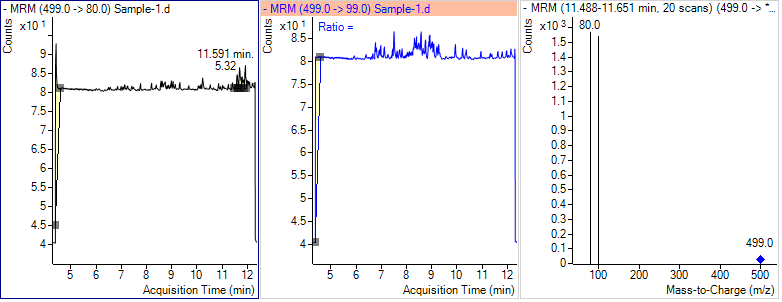


*Figure S11 L-PFOS in field blank sample*
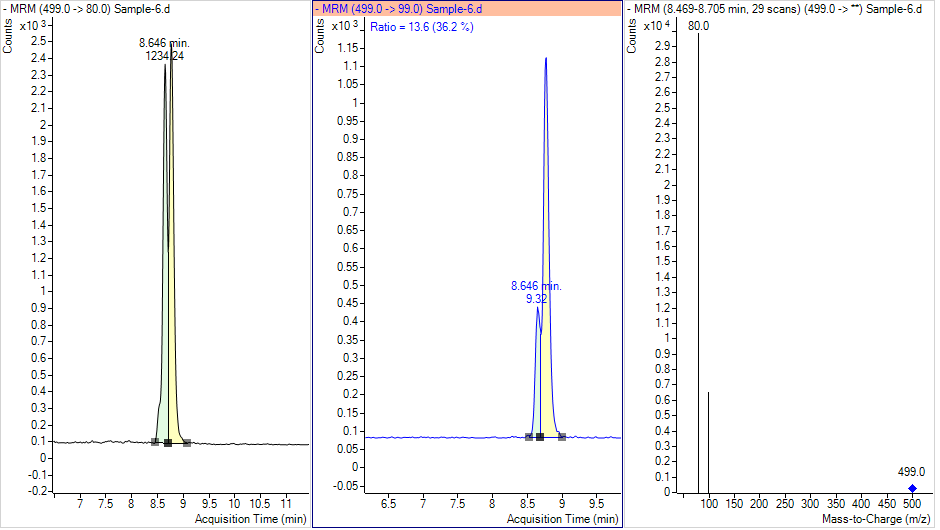


Figure S12 Br-PFOS in sample


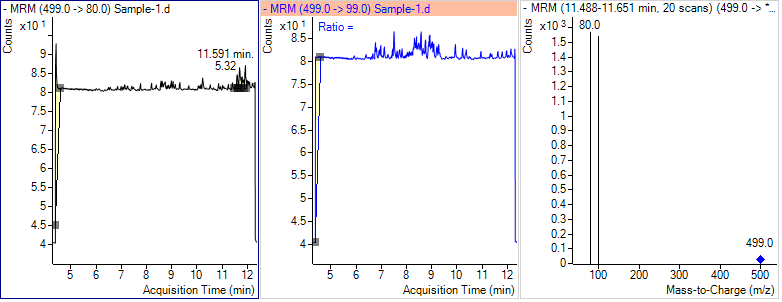


Figure S13 Br-PFOS in field blank sample


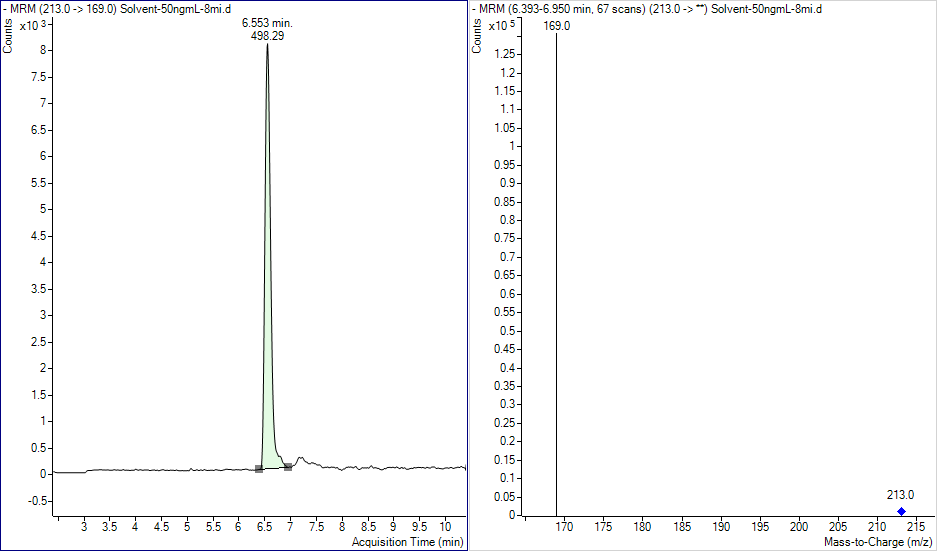


Figure S14 PFBA standard


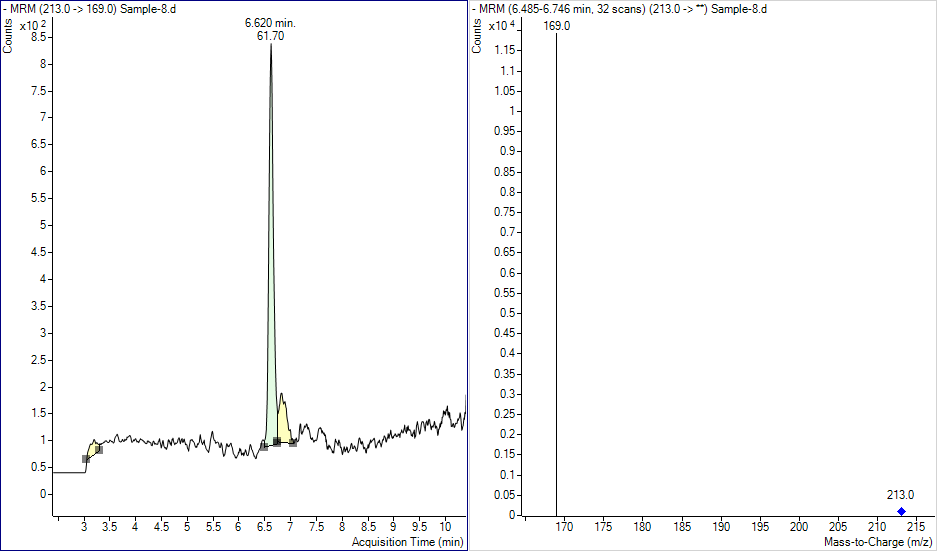


Figure S15 PFBA in sample


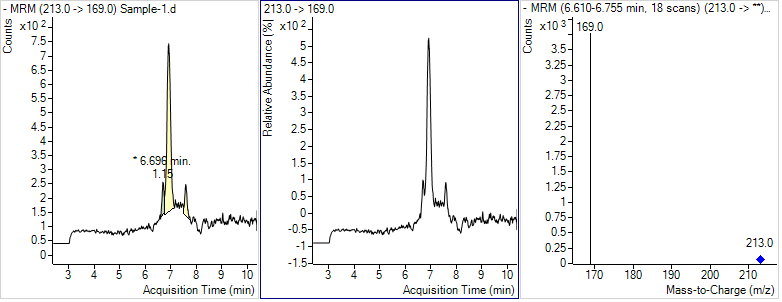


Figure S16 PFBA in field blank


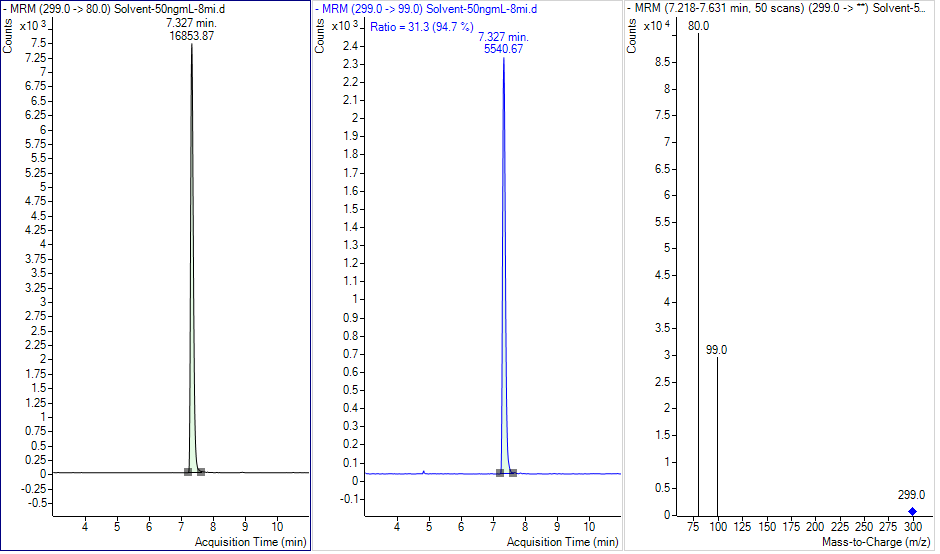


Figure S17 PFBS standard


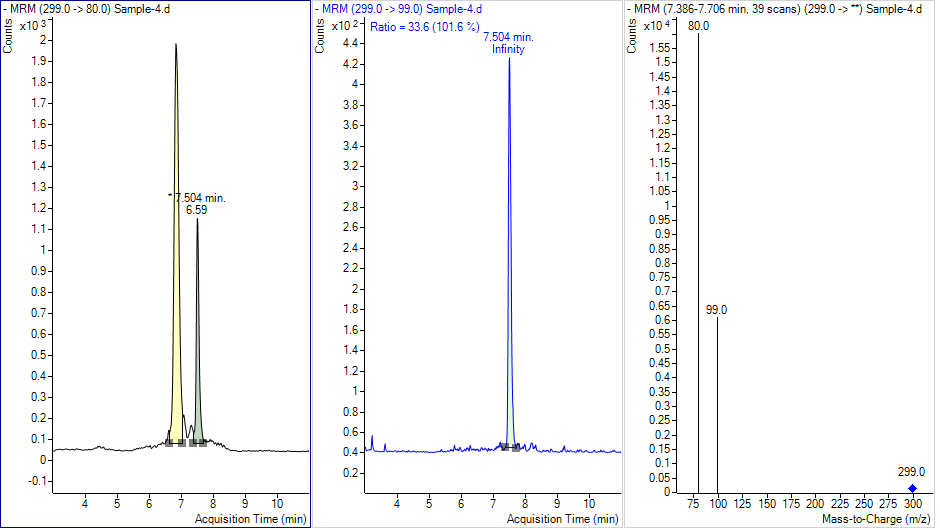


Figure S18 PFBS in sample


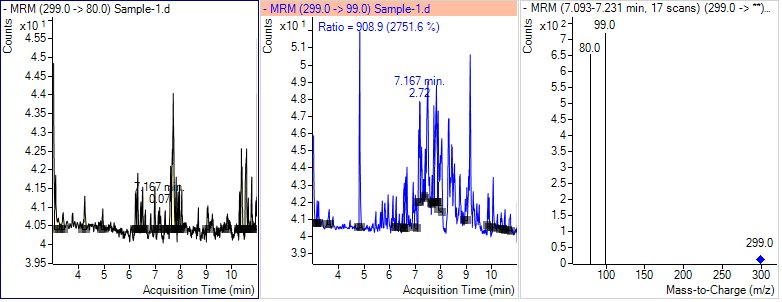


Figure S19 PFBS in field blank


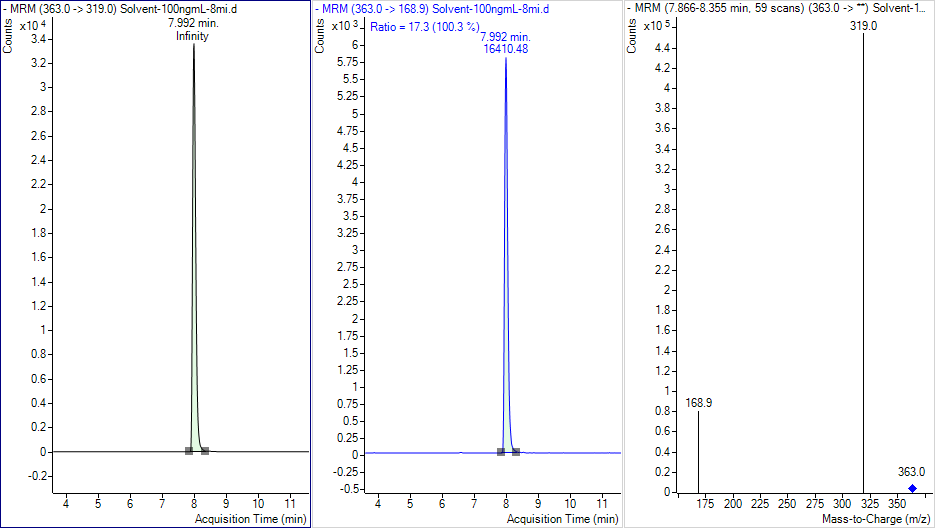


Figure S20 PFHpA standard


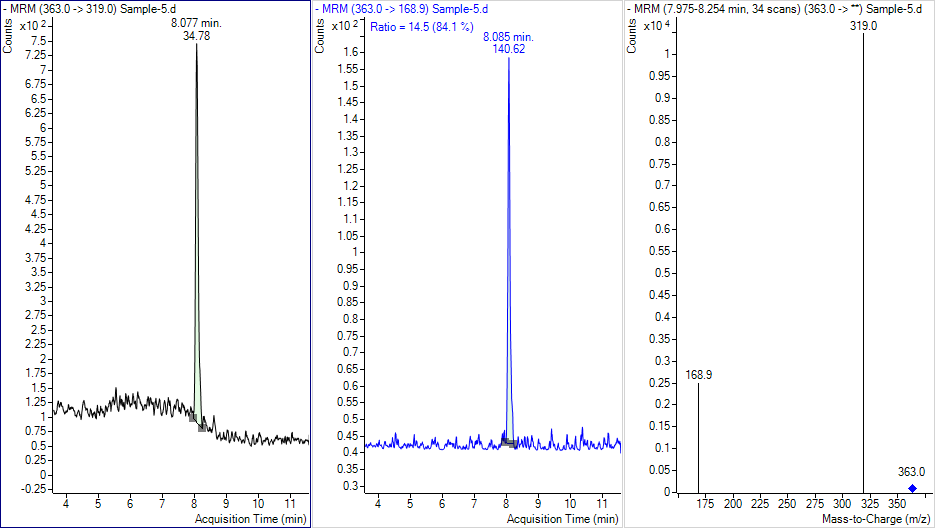


Figure S21 PFHpA in sample


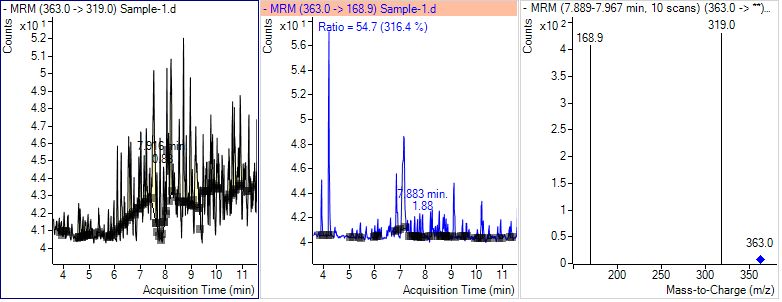


Figure S22 PFHpA in field blank


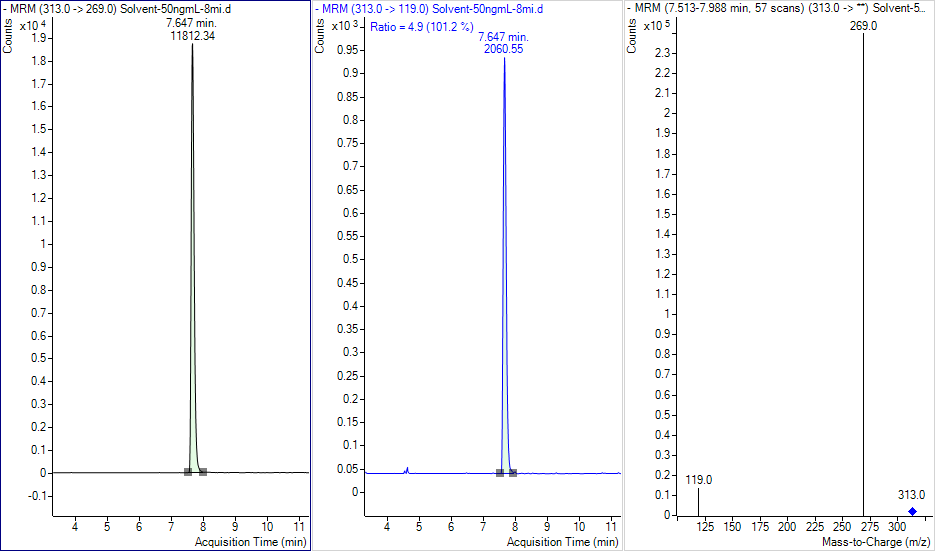


Figure S23 PFHxA standards


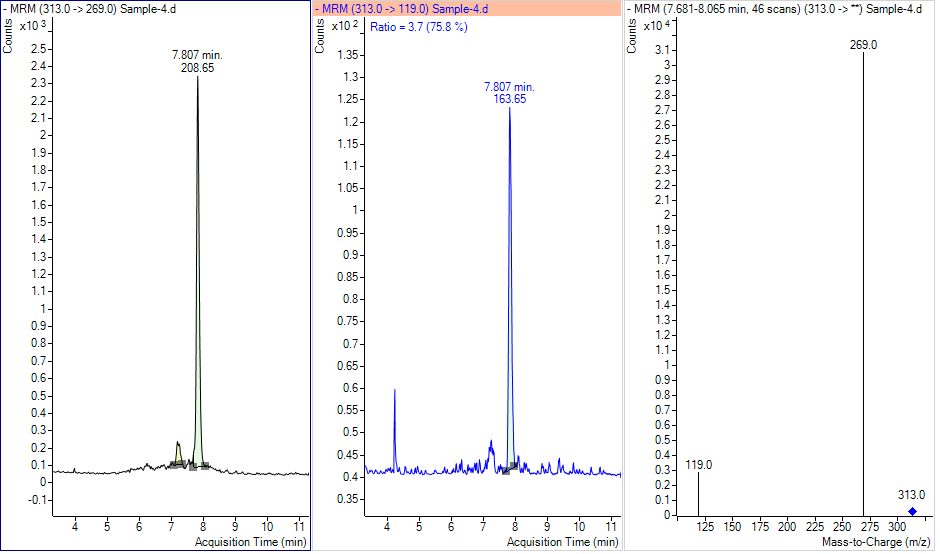


Figure S24 PFHxA in sample


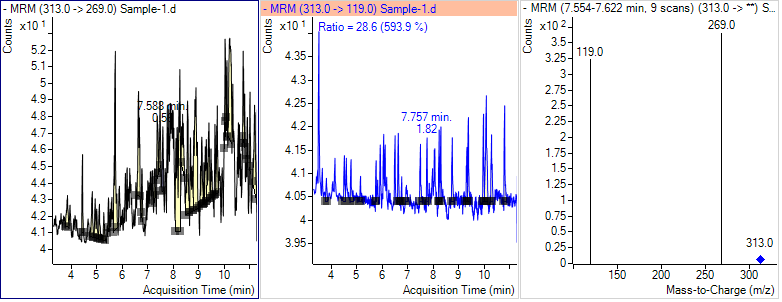


Figure S25 PFHxA in field blank


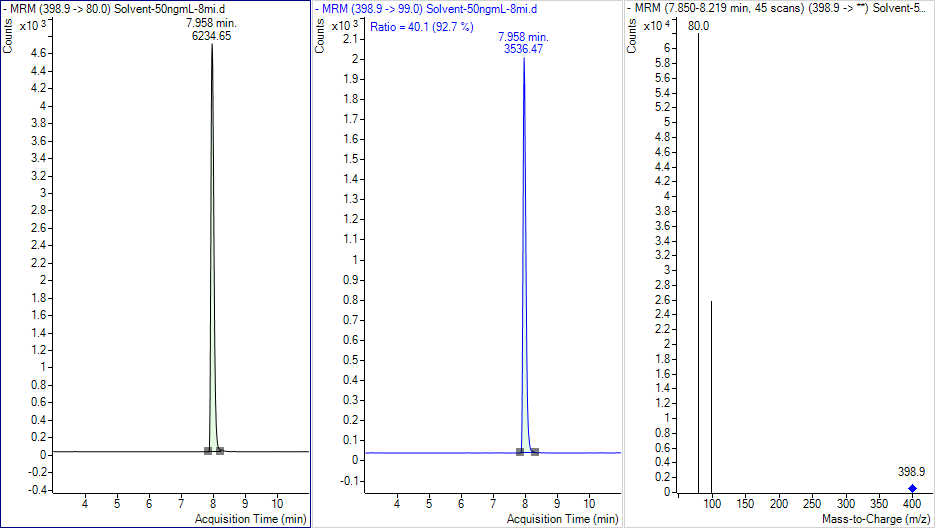


Figure S26 PFHxS standard


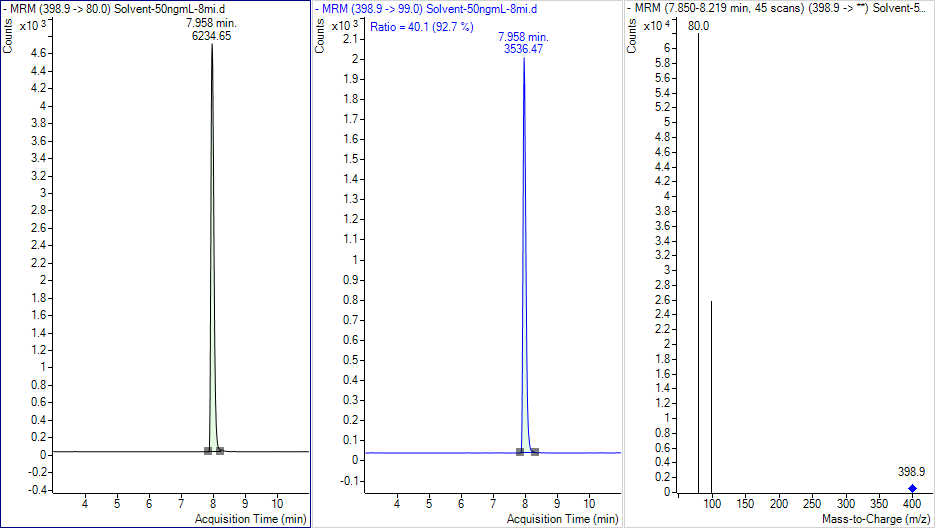


Figure S27 PFHxS in sample


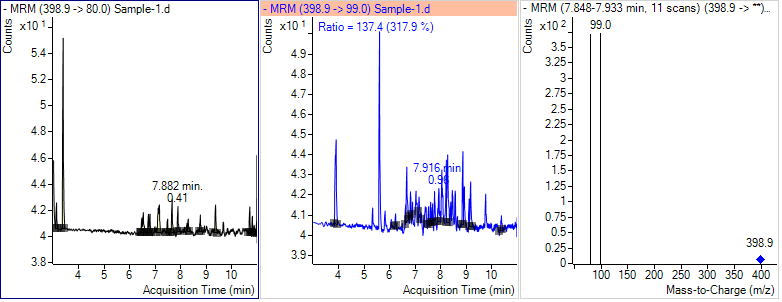


Figure S28 PFHxS in field blank


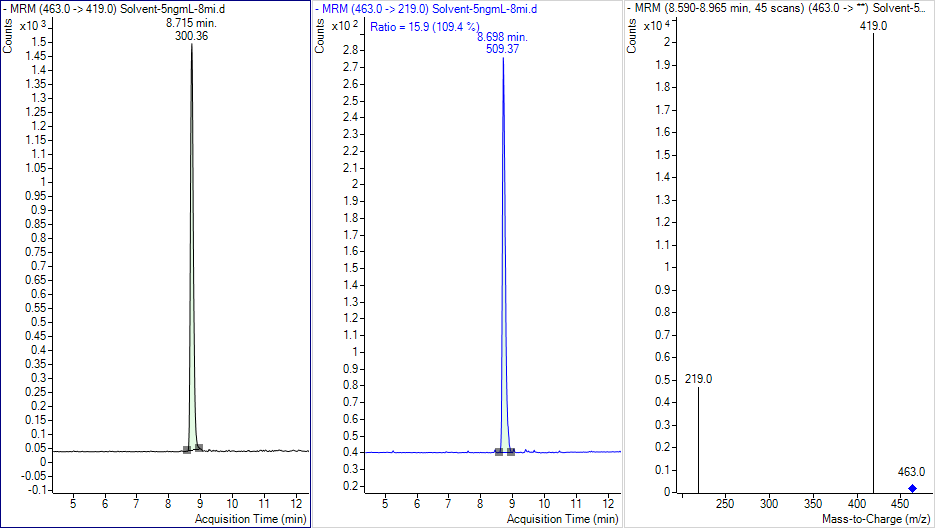


Figure S29 PFNA standard


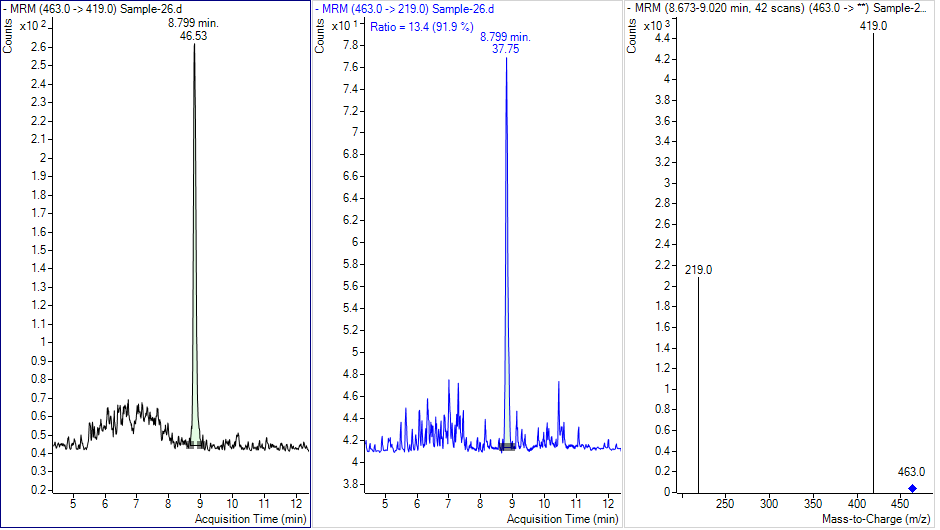


Figure S30 PFNA Sample


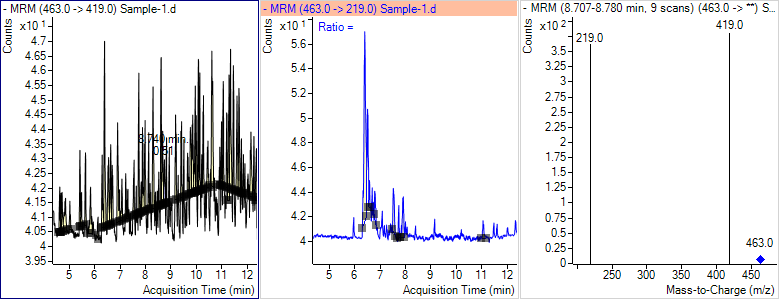


Figure S31 PFNS in field blank

References

Joerss H, Apel C, Ebinghaus R (2019) Emerging per-and polyfluoroalkyl substances (PFASs) in surface water and sediment of the North and Baltic Seas Sci Total Environ 686:360-369

Sánchez-Avila J, Meyer J, Lacorte (2010) Spatial distribution and sources of perfluorochemicals in the NW Mediterranean coastal waters (Catalonia, Spain) Environ Poll 158:2833-2840

Skaar JS, Ræder EM, Lyche JL, Ahrens L, Kallenborn R (2019) Elucidation of contamination sources for poly-and perfluoroalkyl substances (PFASs) on Svalbard (Norwegian Arctic) Environ Sci Poll Res 26:7356-7363

Yamashita N, Kannan K, Taniyasu S, Horii Y, Petrick G, Gamo T (2005) A global survey of perfluorinated acids in oceans Mar Poll Bull 51:658-668

Zheng H,  Wang F,  Zhao Z,  Ma Y,  Yang H,  Lu Z,  Cai Z,  Cai M (2017) Distribution profiles of per-and poly fluoroalkyl substances (PFASs) and their re-regulation by ocean currents in the East and South China Sea Mar Poll Bull 125:481-486
